# Why are the phenotypes of TRAF6 knock-in and TRAF6 knock-out mice so different?

**DOI:** 10.1371/journal.pone.0263151

**Published:** 2022-02-14

**Authors:** Tsvetana Petrova, Kyle Bennett, Sambit Nanda, Sam Strickson, Cheryl L. Scudamore, Alan R. Prescott, Philip Cohen

**Affiliations:** 1 MRC Protein Phosphorylation and Ubiquitylation Unit, School of Life Sciences, University of Dundee, Dundee, United Kingdom; 2 Division of Cell Signalling, School of Life Sciences, University of Dundee, Dundee, United Kingdom; 3 Exepathology, Exeter, Devon, United Kingdom; 4 Dundee Imaging Facility and Division of Cell Signalling and Immunology, School of Life Sciences, University of Dundee, Dundee, United Kingdom; Universite Paris-Saclay, FRANCE

## Abstract

The expression of TNF-Receptor Associated Factor 6 (TRAF6) is essential for many physiological processes. Here we studied the phenotype of TRAF6[L74H] knock-in mice which are devoid of TRAF6 E3 ligase activity in every cell of the body, but express normal levels of the TRAF6 protein. Remarkably, TRAF6[L74H] mice have none of the phenotypes seen in TRAF6 KO mice. Instead TRAF6[L74H] mice display an entirely different phenotype, exhibiting autoimmunity, and severe inflammation of the skin and modest inflammation of the liver and lungs. Similar to mice with a Treg-specific knockout of TRAF6, or mice devoid of TRAF6 in all T cells, the CD4^+^ and CD8^+^ T cells in the spleen and lymph nodes displayed an activated effector memory phenotype with CD44^high^/CD62L^low^ expression on the cell surface. In contrast, T cells from WT mice exhibited the CD44^low^/CD62L^high^ phenotype characteristic of naïve T cells. The onset of autoimmunity and autoinflammation in TRAF6[L74H] mice (two weeks) was much faster than in mice with a Treg-specific knockout of TRAF6 or lacking TRAF6 expression in all T cells (2–3 months) and we discuss whether this may be caused by secondary inflammation of other tissues. The distinct phenotypes of mice lacking TRAF6 expression in all cells appears to be explained by their inability to signal via TNF Receptor Superfamily members, which does not seem to be impaired significantly in TRAF6[L74H] mice.

## Introduction

The expression of TNF-Receptor (TNFR) Associated Factor 6 (TRAF6) is essential for many physiological processes. TRAF6 knock-out (KO) mice are born at sub-Mendelian ratios, most TRAF6 KO embryos dying between embryonic day 14 and birth, for reasons that include defects in neural tube closure [1]. The TRAF6 KO mice that are born alive do not develop any guard-hair follicles and have modified sebaceous glands [2]. They also have deformed bones and their teeth do not erupt due to osteopetrosis (abnormal thickening of the bone) caused by the absence of osteoclasts [3] or to the formation of osteoclasts incapable of resorbing bone [4]. This phenotype may explain why TRAF6 KO mice are runted and die within a few weeks of birth.

The expression of the TRAF6 protein is also essential for the development of the immune system. TRAF6 KO mice lack lymph nodes [3] and have defective maturation, activation and development of dendritic cells, and lack a major population of CD4^+^CD8a^-^ splenic dendritic cells [5]. Mice with a B-cell-specific knock-out of TRAF6 have reduced numbers of B cells in their bone marrow and spleen [6]. The expression of TRAF6 is also essential for signal transduction and cytokine production by Interleukin-1 (IL-1) and Toll-Like Receptors (TLRs) [3, 4, 7].

TRAF6 possesses E3 ubiquitin ligase activity, which is associated with its Really Interesting New Gene (RING) domain [8]. For many years, it was widely accepted that the E3 ligase activity of TRAF6 was essential for IL-1 signal transduction, as well as signaling by the TNF superfamily member RANKL (also called TRANCE), which activates the TNFR superfamily member RANK to control the development of osteoclasts. However, these conclusions were based on experiments in which IL-1 or RANKL signaling could be restored in embryonic fibroblasts or monocytes from TRAF6 KO mice by the re-expression of WT TRAF6, but not by the E3 ligase-inactive TRAF6[C70A] mutant [9–11]. In contrast, we found that IL-1β-dependent signal transduction and production of IL-8 could be restored to TRAF6 KO human HEK293 cells and human HACAT cells by the re-expression of two E3 ligase-inactive mutants of TRAF6, as well as by an N-terminally truncated TRAF6 entirely lacking the RING domain [12]. Similarly, other investigators found that TRAF6 mutants lacking the RING domain restored RANKL-dependent differentiation to multinuclear osteoclasts when they were re-expressed in TRAF6 KO splenocytes [13].

These findings prompted us to generate a TRAF6 knock-in mouse (the TRAF6[L74H] mutant) in which the E3 ligase activity of TRAF6 was inactivated, but not its expression, so that all the other functional domains of the protein were intact, including the C-terminal TRAF domain, which interacts with Pro-Xaa-Glu motifs (where Xaa is any amino acid) present in RANK, other TNFR family members, such as CD40, or proteins that interact with the IL-1R and Toll-Like Receptor signaling complexes, such as IRAK1 and IRAK2 [14]. The ligand-induced interaction of TRAF6 with these receptors/receptor-associated proteins triggers the trimerization of TRAF6 and the formation of even higher ordered structures, which activates its E3 ligase activity and permits it to interact with other proteins [9, 14, 15]. Studies with bone marrow-derived macrophages (BMDM) from the TRAF6[L74H] mice revealed that, up to one hour, TLR and RANK signaling was only reduced modestly [12]. Moreover, and consistent with RANK signaling being relatively unimpaired, the teeth of the TRAF6[L74H] mice erupted normally, and the histological structure of their long bones was similar to WT mice [12]. However, the secretion of proinflammatory cytokines induced by prolonged stimulation of BMDM with TLR ligands that signal via MyD88 was greatly decreased [12].

The finding that the E3 ligase activity of TRAF6 was not essential for IL-1R, TLR or RANK signalling in some cells and *in vivo* raised the question of how many of the essential roles of TRAF6 require its catalytic E3 ligase activity. Here, we present a more detailed analysis of the phenotype of the TRAF6[L74H] mice. Remarkably, we found that these mice do not have any of the phenotypes displayed by the TRAF6 KO mice, but instead develop an equally severe but entirely different phenotype.

## Materials and methods

### Generation and maintenance of mice

The generation of TRAF6[L74H] knock-in mice has been described [12]. TRAF6[L74H] mice were back-crossed for 6 generations to C57Bl6/J mice (Charles River Laboratories). Mice were provided with free access to food (R&M3 pelleted irradiated diet) and water. Animals were kept in individually ventilated cages at 21°C, 45–65% relative humidity and a 12 h/12 h light/dark cycle under specific-pathogen–free conditions in accordance with UK and European Union regulations. Ear thickness was measured post-mortem using a Mitutoyo 7301 dial thickness gauge (Mitutoyo, Kawasaki, Japan). Experiments on mice were approved by the University of Dundee Ethical Review Committee under a UK Home Office project license.

### Preparation and histopathological analysis of tissues

Mice were euthanized with increasing concentration of CO_2_. The skin, tail, kidneys, liver, and lungs were removed and fixed for 48–72 h in 10% neutral buffered formalin. Tissues were processed and stained with haematoxylin and eosin (H&E) as described [16]. For immunohistochemistry (IHC), antigen retrieval was performed using heat-induced epitope retrieval (HIER). Sections were treated at full pressure with Access Retrieval Unit (Menarini diagnostics, UK) in sodium citrate buffer (pH 6.0) for 90 sec at 125°C and then rinsed in Tris/HCl Tween buffer (pH 7.5). The sections were treated for 5 min at 21°C with 3% (v/v) hydrogen peroxide in phosphate buffered saline to quench endogenous peroxidase activity. After washing twice with TRIS/HCl Tween buffer (pH 7.5), the sections were incubated for 30 min at 21°C with anti-CD3 (Dako) at 1:100, anti-PAX5 (Abcam) at 1:500, anti-IBA-1 (Alpha Lab) at 1:1500 and anti-p21 (Abcam) at 1:500 dilution. Sections stained with isotype control antibodies were used as negative controls. The sections were washed with Tris/HCl Tween buffer (pH 7.5) to remove the excess primary antibody, then incubated with EnVision+ system HRP Labelled Polymer anti-rabbit secondary antibody (Dako) for 30 min at 21°C. The sections were washed with Tris/HCl Tween buffer pH 7.5 to remove excess labelled polymer from the sections, followed by two 5 min incubations with 3,3’-diaminobenzidine (DAB) substrate-chromogen (EnVision+ System, Dako) and two 5 min rinses with distilled water. Tissues were counterstained using Gill’s haematoxylin and mounted using DPX mounting media (Cellpath) and coverslips for long-term storage.

The tissue sections were assessed by a veterinary pathologist (C.S.) blinded to the genotype of the mice in the different cohorts. Pathological changes in H&E-stained sections of the skin, liver, lungs and thymus were scored using a non-linear semi-quantitative grading system from 0 to 5, where 0 = no significant change and 5 = whole organ or tissue affected. The frequency of IHC stained cells in the tissue were scored using a non-linear semi-quantitative grading system -, +, ++, +++) [17]. Photomicrographs were captured using Nanozoomer software from whole slide image scans prepared using Hamamatsu Nanozoomer HT slide scanner. For preparation of the figures, images were obtained and processed using an Olympus CellSens Standard and resized using Adobe Photoshop.

Terminal deoxynucleotidyl transferase dUTP nick end labelling (TUNEL) staining was performed using the Click-iT^™^ Plus TUNEL Assay for In Situ detection of Apoptosis using Alexa Fluor^™^ 488 dye (ThermoFisher) according to the manufacturer’s instructions. 15 images per mouse were acquired using a Zeiss 710 Xenon microscope and analyzed using Volocity 3D Image Analysis Software. TUNEL positive cells were quantified as the percentage of the total number of cells (DAPI-positive).

### Flow cytometry analysis

The antibodies used for flow cytometry analysis and their sources are summarized in Table 1. Single-cell suspensions were prepared from the spleen and lymph nodes. Red blood cell lysis, cell counting, and antibody staining were performed as described [16]. Dead cells were excluded by staining samples with 0.5 μg/ml of DAPI (BioLegend) or using the eBioscience^™^ Fixable Viability Dye eFluor^™^ 450 (FVD) (ThermoFischer) according to the manufacturer’s instruction. Cells stained with FVD were fixed using the IC Fixation Buffer (ThermoFisher) for 15 minutes at 4°C. Red blood cell lysis was not carried out for analysis of the erythrocyte population of the spleen. For detection of the FOXP3 and Bcl-6 transcription factors, following surface staining, samples were fixed and permeabilised using the FOXP3 transcription factor staining kit (Thermo fisher cat number 00-5523-00) in accordance with the manufacturer’s instructions. Data were collected using BD FACSCanto or BD LSRFortessa II and BD FACSDiva software (BD Bioscience) and the results analyzed by FlowJo software. Doublets were excluded by gating for Forward Scatter-Area (FSC-A) and Forward Scatter-Width (FSC-W), whereas DAPI^-ve^ or FVD^-ve^ cells were gated for further analysis excluding dead cells.

**Table 1:** List of antibodies used for the flow cytometry analysis of various immune cell populations.

Antibodies	Fluorophore	Clone	Source
CD115	APC	AFS98	Biolegend
CD11B	PE/CY7	M1/70	Biolegend
CD11C	PE/DAZZLE594	N418	Biolegend
CD45	BV510	30-F11	Biolegend
CX3CR1	PE	SA011F11	Biolegend
I-A/I-E (MHC II)	Alexafluor700	M5/114.15.2	Biolegend
LY-6C	FITC	HK1.4	Biolegend
LY-6C	BV421	HK1.4	Biolegend
LY-6G/LY-6C (GR-1)	PERCP/CY5.5	RB6-8C5	Biolegend
NK1.1	APC/CY7	PK136	Biolegend
TCRb	PERCP/CY5.5	H57-597	Biolegend
TCRb	FITC	H57-597	Biolegend
B220	FITC	RA3-6B2	Biolegend
CD4	APC-eFluor 780	GK1.5	ThermoFisher/Invitrogen
CD8	PE/CY7	53–6.7	Biolegend
CD62L	FITC	MEL-14	Biolegend
CD44	PE	IM7	Biolegend
CD19	APC	6D5	Biolegend
CD21/35	FITC	7G6	BD Pharmigen
CD23	PE/CY7	B3B4	Biolegend
IgD	Alexafluor700	11-26c.2a	Biolegend
IgM	APC/CY7	RMM-1	Biolegend
CD138	PE	281–2	Biolegend
Ly-76 (Erythroid cell)	PECy7	Ter 119	Biolegend
CXCR5	BV605	L138D7	Biolegend
PD1	BV421	29F.1A12	Biolegend
BCL-6	PE	BCL-DWN	ThermoFisher/Invitrogen
ICOS (CD278)	APC	C398.4A	ThermoFisher/Invitrogen
CD25	PE/CY7		Biolegend
FOXP3	APC		ThermoFisher/Invitrogen ThermoFisher/Invitrog

### Other methods

Cytokines were measured using Luminex-based Bio-Plex Mouse Grp 1 Cytokine 23 plex (Bio-Rad Laboratories) or individual Bio-Plex cytokine kits following the manufacturer’s instructions. Mouse immunoglobulin isotypes were measured as described [18] using MILLIPLEX MAP Mouse Immunoglobulin Isotyping Magnetic Bead Panel (MGAMMAG-300K, for measurements of IgG1, IgG2a, IgG2b, IgG3, IgA and IgM) and MILLIPLEX MAP Mouse IgE Single Plex Magnetic Bead Kit (MGAMMAG-300E, for measurement of IgE) following manufacturer’s instruction. Autoantibodies to double stranded DNA (dsDNA) and antinuclear antibodies (ANA) (total Ig; Alpha Diagnostics International) were measured as described before by ELISA [19].

### Statistical analysis

Data were analysed using GraphPad Prism 9 software. Figures were made using GraphPad Prism, Adobe Illustrator and FlowJo. The distribution was determined using the Shapiro–Wilk normality test. Pair-wise comparison of parametric and nonparametric data was done using the unpaired t test with Welch’s correction or unpaired Mann-Whitney test, respectively.

## Results

### Phenotypic analysis of TRAF6[L74H] mice

**Fig 1:**
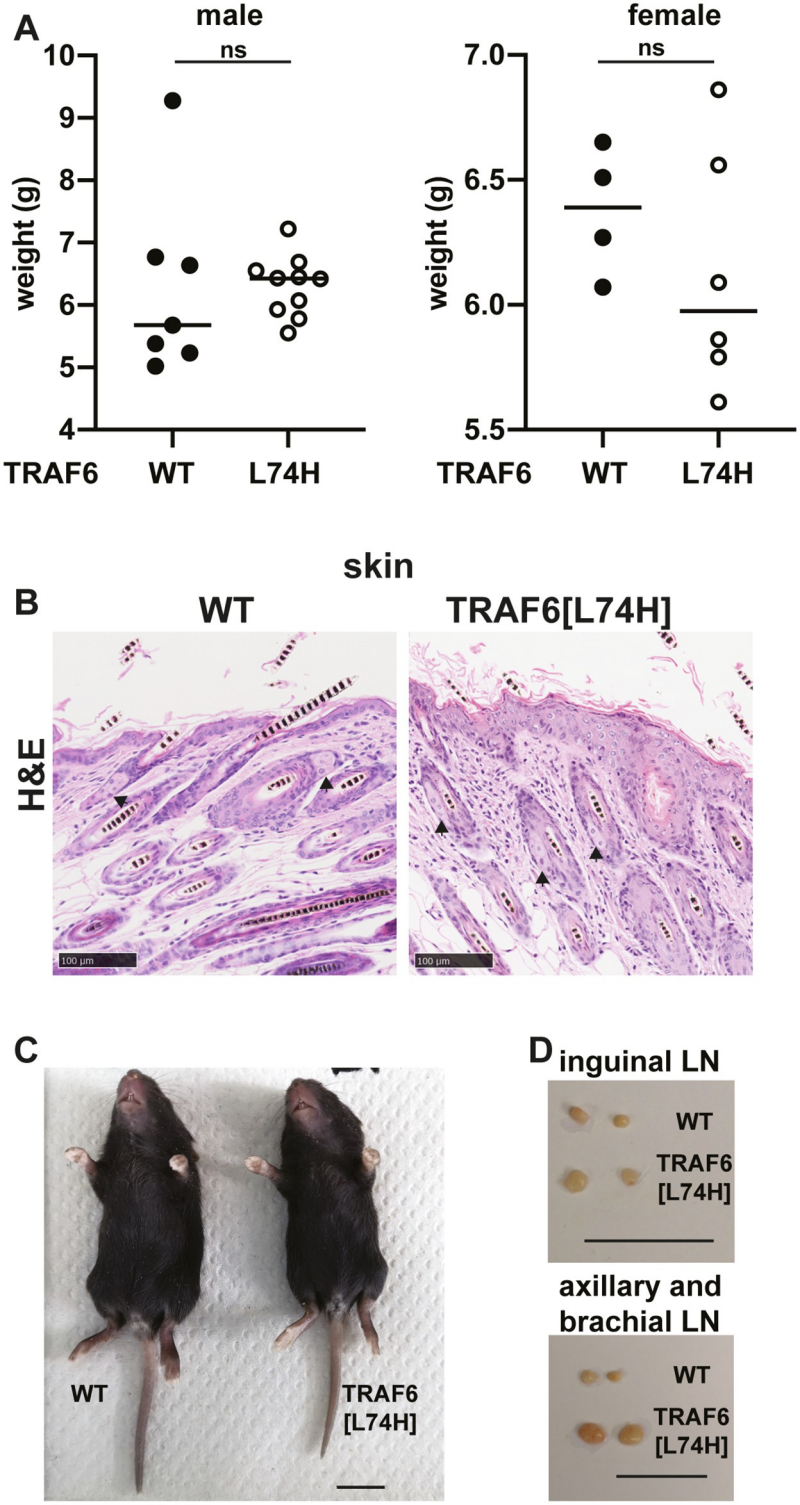
TRAF6[L74H] mice phenotype. (A) Body weight of 16 days old male WT (n = 6) and TRAF6[L74H] (n = 8) mice (left-hand panel) and female WT (n = 4) and TRAF6[L74H] mice (n = 6) (right-hand panel). (B) Representative hematoxylin-eosin (H&E) staining image of the skin from WT and TRAF6[L74H] mice. Arrows show sebaceous glands. (C) Representative image of a 16-day old WT and TRAF6[L74H] male mice. (D) As in C, except images show enlarged inguinal (upper panel) and axillary and brachial lymph nodes (lower panel). Symbols in A represent individual biological replicates. Statistical significance was calculated using the Mann-Whitney test (left-hand panel) and unpaired t test with Welch’s correction. Individual values, descriptive statistics and results from the statistical analysis are provided in S1 File.

In contrast to TRAF6 KO mice (see Introduction), the TRAF6[L74H] mice were born at near normal Mendelian frequencies (51.25% heterozygous, 28.13% wildtype (WT), 20.63% homozygous), and their weight up to 16 days did not differ significantly from WT mice (Fig 1A). Unlike TRAF6 KO mice, the TRAF6[L74H] mice developed sebaceous glands of normal morphology and number (Fig 1B). TRAF6 KO mice lack guard-hair follicles, a phenotype that can be detected by the absence of hair on their tails; in contrast WT mice only lack hair at the tips of their tails [2]. The tails of TRAF6[L74H] mice showed a similar distribution of hair to WT mice, and they also lacked the kink at the end of the tail (Fig 1C), which is characteristic of TRAF6 KO mice [2]. TRAF6 KO mice lack lymph nodes, but the lymph nodes of TRAF6[L74H] mice were not only present but enlarged compared to WT littermates (Fig 1D).

### Splenic cell numbers in TRAF6[L74H] mice

**Fig 2:**
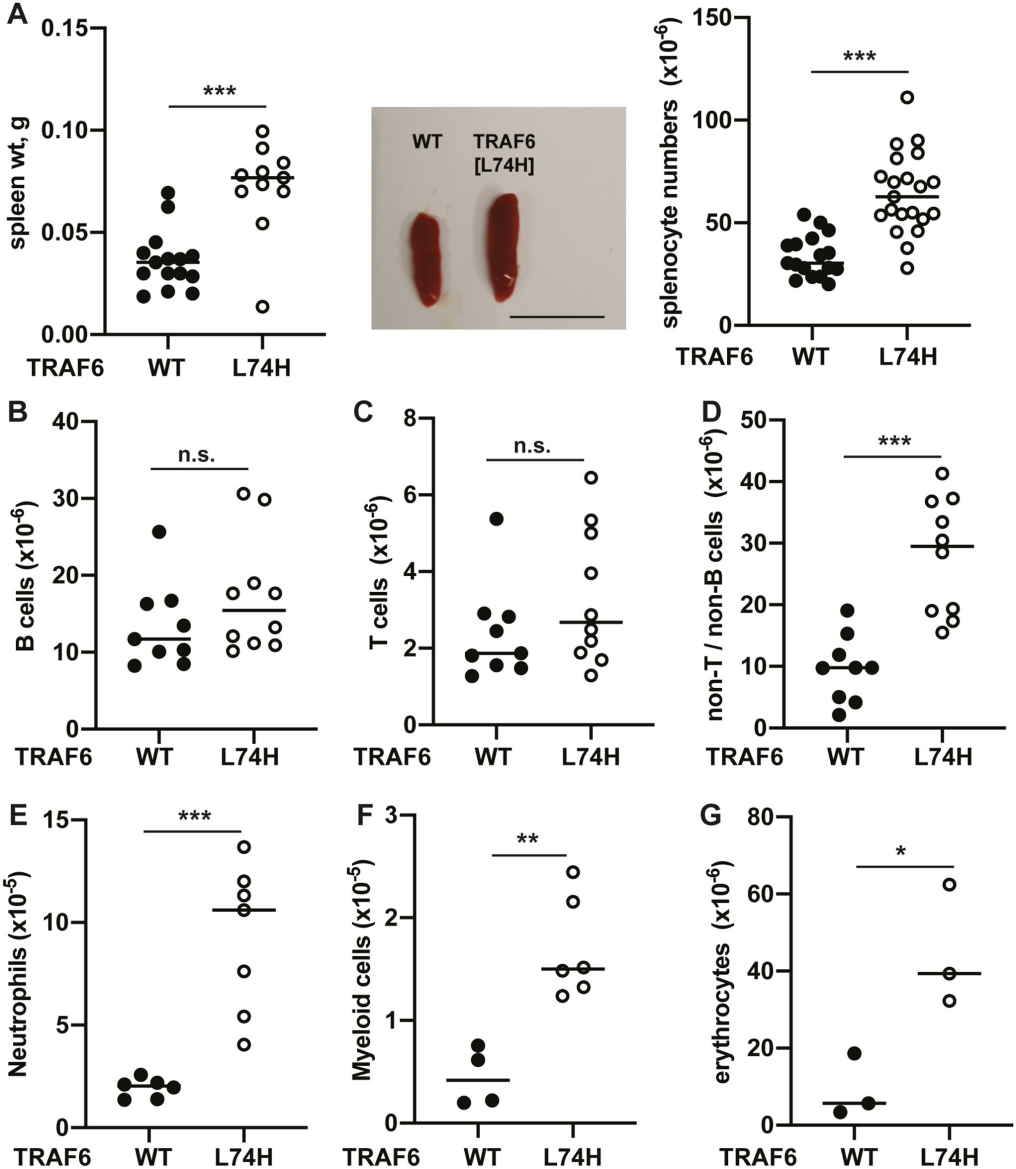
Characterization of splenic immune cell populations in TRAF6[L74H] mice. (A) Spleen weight (left panel) and representative images of spleen enlargement (middle panel) and total splenocyte numbers of 16 days old WT (n = 15–17) and TRAF6[L74H] (n = 11–21) mice, scale bar = 1 cm. (B-D) Immune cell populations in the spleen of 16 days old WT and TRAF6[L74H] mice were analyzed by flow cytometry. Total numbers of (B) B-cells, (C), T-cells, (D) and non-T/B cells in spleens of WT (n = 9) and TRAF6[L74H] (n = 10) mice are shown. (E-G), as in B-D, except that (E) neutrophils from WT (n = 6) and TRAF6[L74H] (n = 7) mice (F) Gr-1^-^CD11b^+^ myeloid cells from WT (n = 4) and TRAF6[L74H] (n = 6) mice or (G) erythrocytes WT (n = 3) and TRAF6[L74H] (n = 3) mice were measured. Symbols represent individual biological replicates. Statistical significance between the two genotypes was calculated using the unpaired t-test with Welch’s correction or the Mann-Whitney test. * denotes p<0.05, ** denotes p<0.01, *** denotes p<0.001 and n.s. denotes not significant difference. Individual values, descriptive statistics and results from the statistical analysis are provided in S2 File.

The TRAF6[L74H] mice displayed splenomegaly with an increase in spleen weight and spleen cell number (Fig 2A). We therefore investigated the cell types that were increased. Unlike mice with a B cell-specific knock-out of TRAF6, which have reduced numbers of B cells in their spleen [6], B cell numbers in the spleens of TRAF6[L74H] and WT mice were similar (Fig 2B and S1A Fig upper two panels). The numbers of T cells (Fig 2C and S1A Fig upper two panels), as well as CD4^+^ and CD8^+^ T cell populations (S1A Fig lower two panels and S1B Fig), also did not differ significantly between TRAF6[L74H] and WT mice. The greatest increase in cell number was in the non-T/non-B compartment (Fig 2D), where there were increased numbers of neutrophils (Fig 2E, S1C Fig), Gr-1^-^CD11b^+^ myeloid cells (Fig 2F, S1C Fig), and Ter119^+^ erythrocytes (Fig 2G, S1D Fig). The increased number of erythrocytes was a major reason for the increased number of splenic cells (compare Fig 2A and 2G). The increase in splenic erythrocytes is most likely explained by stress-induced extra-medullary haematopoiesis.

### Inflammation and autoimmunity in TRAF6[L74H] mice

**Fig 3:**
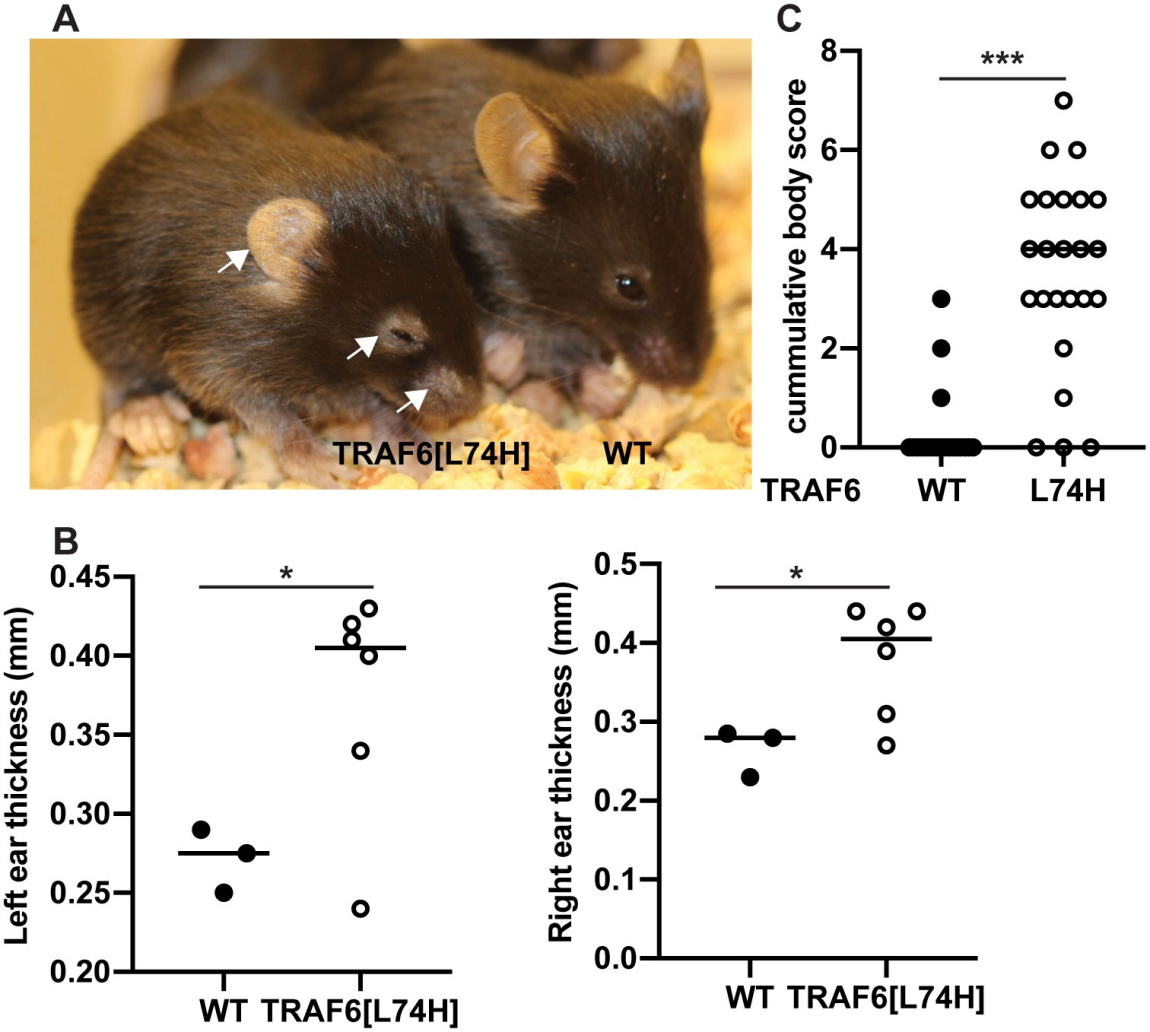
Body condition of TRAF6[L74H] knock-in mice. (A) Representative image showing body condition of a WT and TRAF6[L74H] mouse. (B) Graphs show thickness of the left and right ears of WT (n = 3) and TRAF6[L74H] (n = 6) mice. (C) Cumulative body scores based on skin thickening, ear position, hair loss around the eyes, skin scaling, hunched position, piloerection and face grimace in 16 days old WT (n = 20) and TRAF6[L74H] (n = 24) mice. Symbols represent individual biological replicates. Statistical significance between the two genotypes was calculated using the Mann-Whitney test; * denotes p<0.05 and *** denotes p<0.001. Individual values, descriptive statistics and results from the statistical analysis are provided in S3 File.

In our initial studies, performed using mice that had only been backcrossed once, the animals displayed flaking and scaling of the skin when they were 4–5 weeks old [12]. However, this phenotype developed much earlier in TRAF6[L74H] mice that had been backcrossed six times with flaking and scaling of the skin, particularly around the nose and eyes, being clearly visible after only 16 days (Fig 3A). There was also thickening of the ears by 16 days, which is likely to be caused by hyperkeratinisation and increased cellularity induced by inflammation (Fig 3B). After 16 days, mice also adopted a hunched posture (Fig 3A), indicative of pain and discomfort, which was confirmed by a combination of body and grimace scoring (Fig 3C). This necessitated the TRAF6[L74H] mice being culled routinely at 16 days to comply with the experimental plan agreed with the Veterinary.

**Fig 4:**
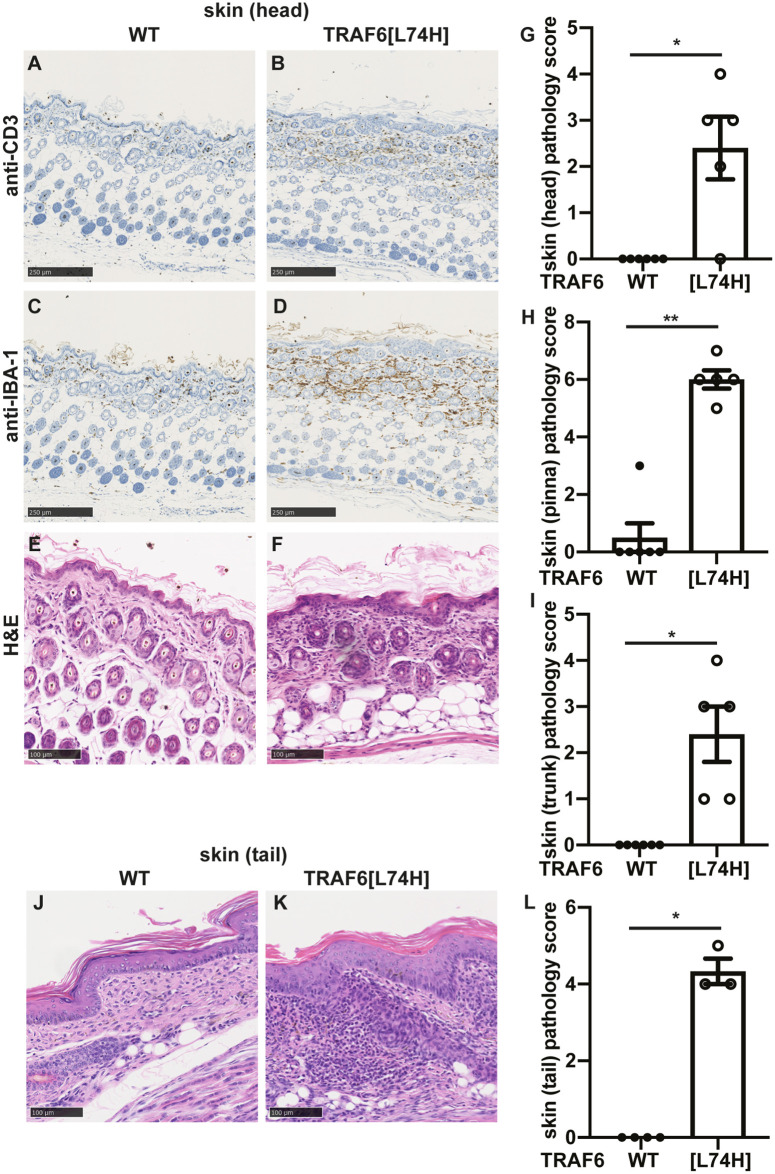
Severe skin inflammation seen in TRAF6[L74H] mice. (A, B) Representative images showing skin sections from WT (A) and TRAF6[L74H] mice processed for immunohistochemistry and stained using anti-CD3 antibody. (C, D) As in A, B except that anti-IBA-1 antibody was used. (E, F) As in A, B except that skin sections from WT (A) and TRAF6[L74H] were stained with haematoxylin and eosin (H&E). (G-I) Bar graphs showing cumulative skin inflammation scores from head (G), pinna (the visible portion of the outer ear) (H) and trunk (I). (J, K) As in E, F, except the representative tail sections are shown. (L) As in G, except that the cumulative pathology score of the tail is shown. Graphs show mean ± SEM. Statistical significance between the two genotypes was calculated using the Mann-Whitney test. * denotes p<0.05 and ** denotes p<0.01. Individual values, descriptive statistics and results from the statistical analysis are provided in S4 File.

Histological examination of the skin of 16-day mice revealed striking infiltration by CD3^+^ T lymphocytes (compare Fig 4A and 4B) in the skin of TRAF6[L74H] mice, but not WT mice. There was also infiltration by circulating macrophages as judged by staining for IBA-1 (Fig 4C and 4D). The immune infiltration was accompanied by changes in the structure of the skin, such as hyperplasia, hyperkeratosis with diffuse dermal inflammatory cell infiltration as assessed by H&E staining (Fig 4E–4I). Similar changes were observed in the skin from the tail (Fig 4J–4L).

The skin sections from the ear were also stained with anti-p21 to examine senescence and subjected to TUNEL staining to examine cell death. p21 staining was enhanced in the outermost layers of the skin from TRAF6[L74H] mice (S2A–S2C Fig), which could be a consequence of the hyperkeratosis and the cause of skin flaking (see Discussion). Enhanced TUNEL staining was observed in the outermost skin sections of just two of the five TRAF6[L74H] mice examined (e.g. S2D Fig), but not in the other three (S2E Fig). Overall, there was not a statistically significant difference between the TRAF6[L74H] and WT mice (S2E Fig).

**Fig 5:**
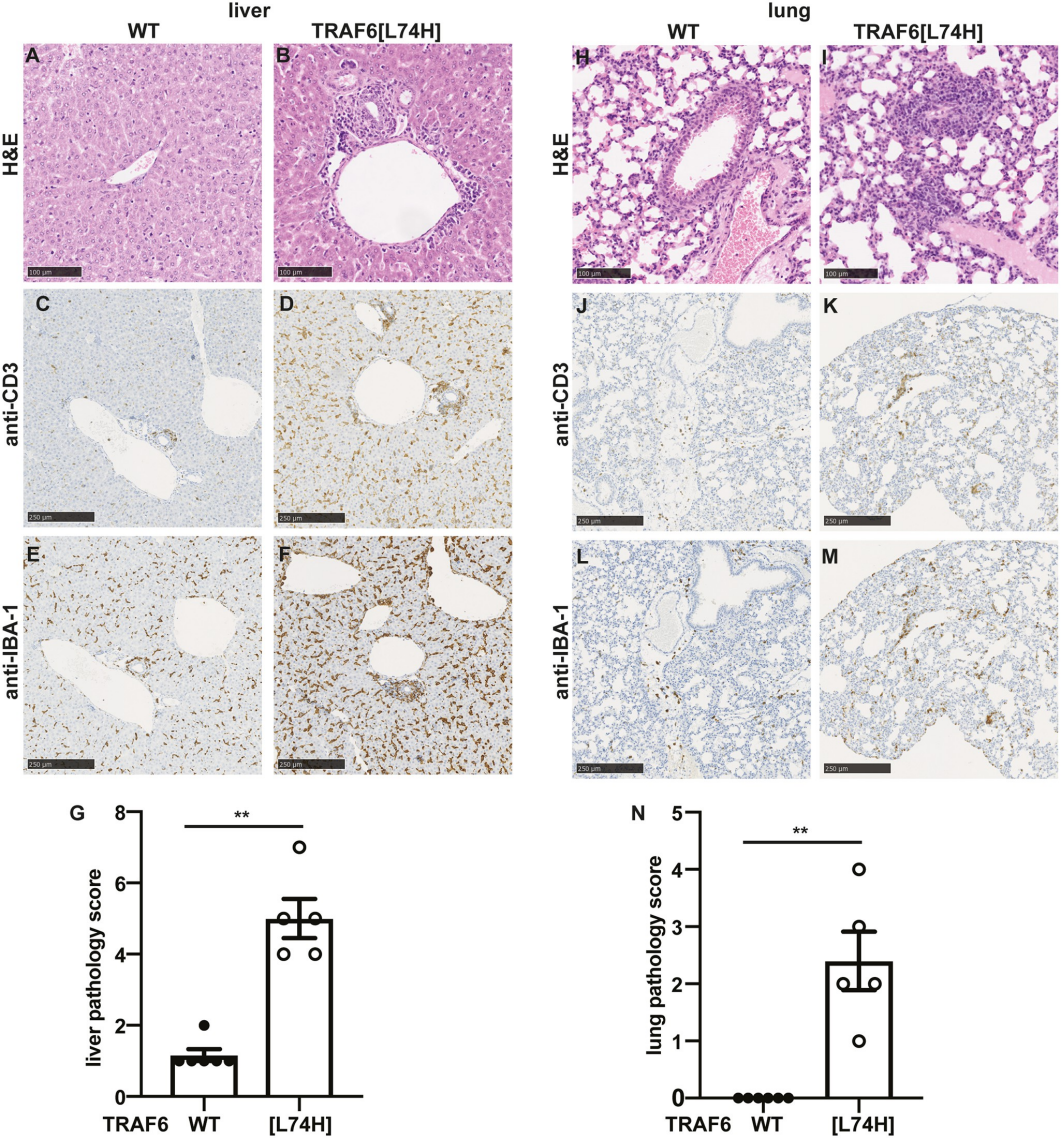
Liver and lung inflammation seen in TRAF6[L74H] mice. (A-F) Representative image showing haematoxylin and eosin (H & E)-stained liver sections from WT (A) and TRAF6[L74H] (B) mice. BV, blood vessel (C, D) As in A, B except that skin sections were processed for immunohistochemistry staining using anti-CD3 antibody. (E, F) As in C, D except that anti-IBA-1 antibody was used. (G) Bar graphs showing liver pathology score. (H-M) As in A-F, except that lung sections are shown. (N) As in G, except lung pathology score is shown. Graphs show mean ± SEM. Statistical significance between the two genotypes was calculated using the Mann-Whitney test. ** denotes p<0.01. Individual values, descriptive statistics and results from the statistical analysis are provided in S5 File.

Histopathological analysis of liver sections from TRAF6[L74H] mice showed mixed inflammatory infiltrates, which were predominantly centered on portal tracts, and in a few animals the inflammation was also perivascular (Fig 5A, 5B and 5G). Increased infiltration of T cells (Fig 5C and 5D), macrophages (Fig 5E and 5F), and B cells (S3A and S3B Fig) were observed in the liver of TRAF6[L74H] mice. The lungs of TRAF6[L74H] mice also had increased perivascular inflammatory infiltrates with extension to peribronchiolar inflammation in some animals (Fig 5H–5N and S3C and S3D Fig). Taken together, these data demonstrated that the TRAF6[L74H] animals displayed multi-organ inflammation.

**Fig 6:**
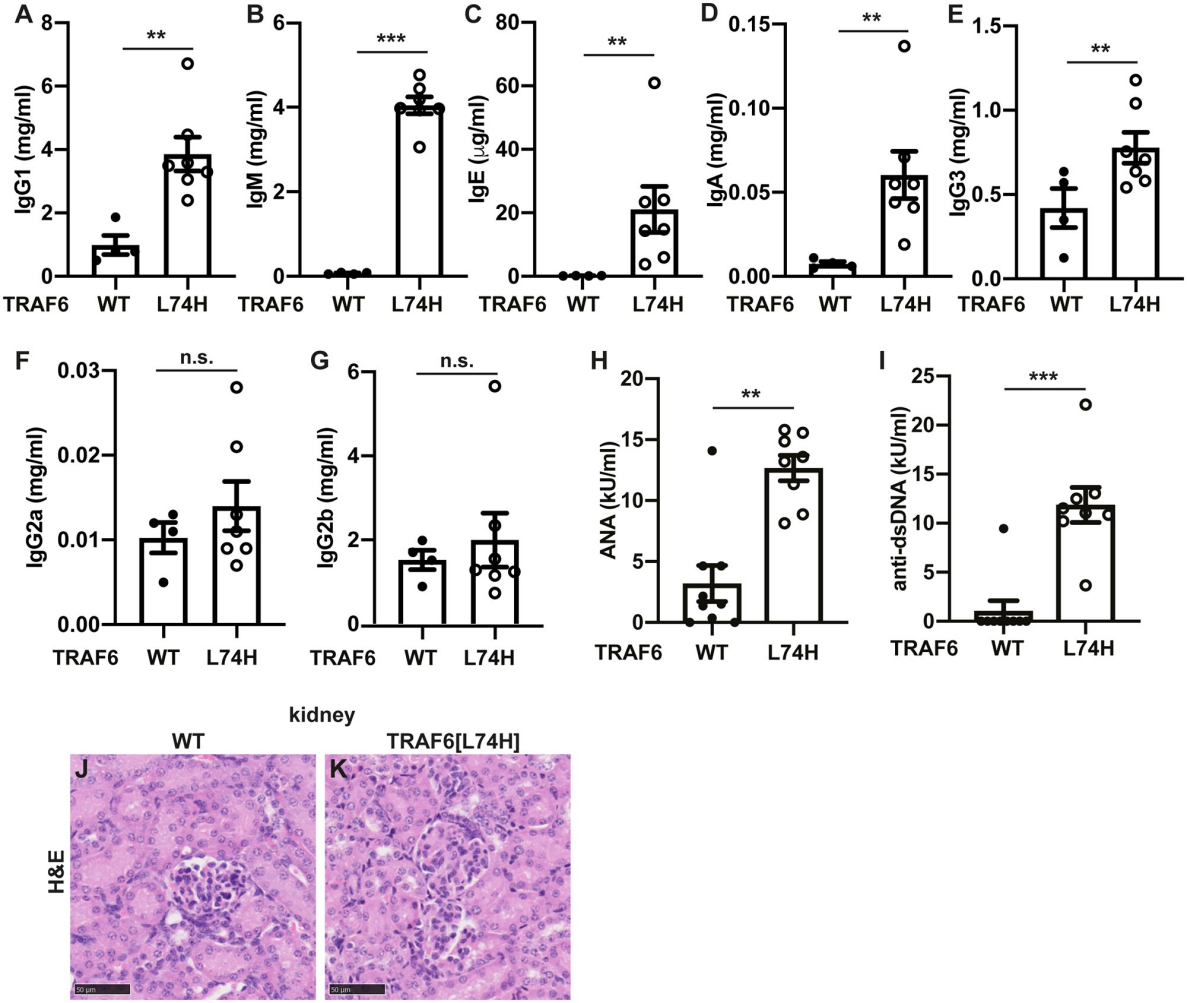
Increased immunoglobulin and autoantibody levels in TRAF6[L74H] mice. Concentrations of IgG1 (A), IgM (B), IgE (C), IgA (D), IgG3 (E), IgG2a (F) and IgG2b (G) in the serum of 16 days old WT (n = 7–9) and TRAF6[L74H] (n = 4–8) mice. (H-I) As in A-G, except that the plots show concentration of ANA (H) and anti-dsDNA (I) antibodies. The error bars show ± SEM. Symbols represent individual biological replicates. Statistical significance between the two genotypes was calculated using the unpaired t-test with Welch’s correction or the Mann-Whitney test. * denotes p<0.05, ** denotes p<0.01, *** denotes p<0.001 and n.s. denotes not significant difference. (J, K) Representative image showing haematoxylin and eosin (H&E)-stained liver sections from WT (J) and TRAF6[L74H] (K) mice. Individual values, descriptive statistics and results from the statistical analysis are provided in S6 File.

Taken together, the phenotypes of the TRAF6[L74H] mice described above were suggestive of an autoimmune/autoinflammatory phenotype. To investigate this possibility further, we examined the levels of immunoglobulins in the serum. These experiments revealed striking increases in the levels of IgG1, IgM, IgE and IgA (Fig 6A–6D), a modest increase in IgG3 (Fig 6E), but no increase in IgG2a or IgG2b (Fig 6F and 6G). The serum of the TRAF6[L74H] mice also had greatly elevated levels of anti-double-stranded (ds) DNA and anti-nuclear antigens (ANA) (Fig 6H and 6I), which are found in a wide range of autoimmune conditions.

Since autoimmunity frequently leads to glomerulonephritis, we studied the kidney pathology, but no glomerulonephritis or any other pathological changes in the kidney were detected in 16-day-old TRAF6[L74H] mice (Fig 6J and 6K), perhaps because the mice were too young for this phenotype to have developed.

### T cell activation in TRAF6[L74H] mice

**Fig 7:**
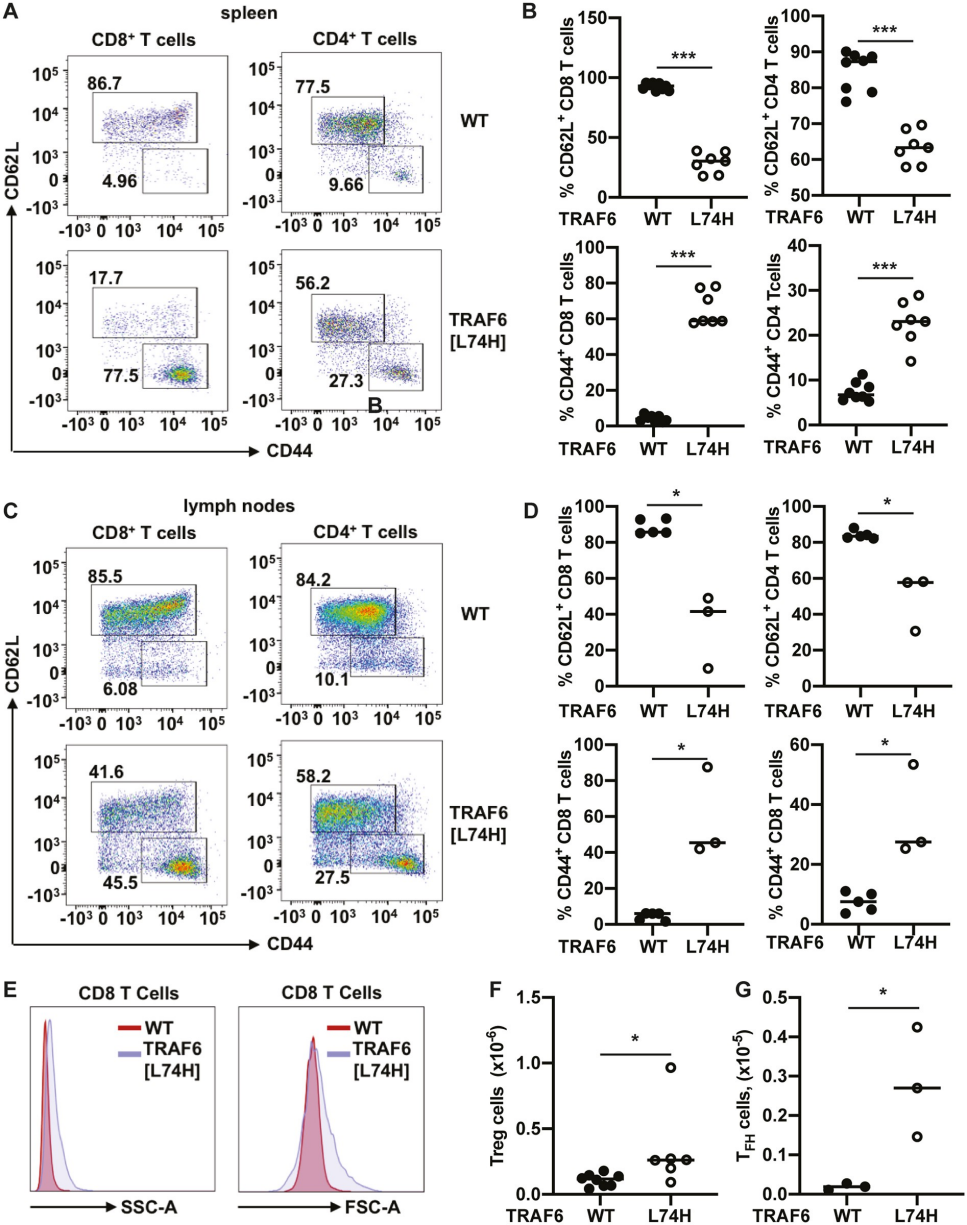
The T cells from TRAF6[L74H] knock-in mice display activated phenotype. (A) Representative flow cytometry plots showing expression of CD62L and CD44 within the CD8 (DAPI^-^TCRβ^+^CD8^+^ population) and CD4 (DAPI^-^TCRβ^+^CD4^+^ population) T cells in the spleens of WT (n = 8) and TRAF6[L74H] (n = 7). (B) Graphs showing percentages of CD62L^+^ CD8 T cells (top left-hand panel), CD62L^+^ CD4 T cells (top right-hand panel), CD44^+^ CD8 T cells (bottom left-hand panel) and CD44^+^ CD8 T cells (bottom right-hand panel). (C, D) As in A and B, except plots show data from lymph nodes of 5 WT and 3 TRAF6[L74H] mice. (E) Representative histograms showing side scatter-area (SSC-A) and forward scatter area (FSC-A) of CD8 T cells (DAPI^-^TCRβ^+^CD8^+^ population) of WT and TRAF6[L74H] mice. (F, G) Total numbers of Treg (F) and T_FH_ cells (G). Symbols represent individual biological replicates. (B, D, G, F) Statistical significance between the two genotypes was calculated using the unpaired t-test with Welch’s (F) correction or the Mann-Whitney test (B, D and G). * denotes p<0.05, *** denotes p<0.001 and n.s. denotes not significant difference. Individual values, descriptive statistics and results from the statistical analysis are provided in S7 File.

To investigate potential reasons for the inflammatory and autoimmune phenotypes of TRAF6[L74H] mice we analysed the splenic T cell populations by flow cytometry. These experiments revealed that both the CD4^+^ and CD8^+^ T cells displayed an activated effector memory phenotype with CD44^high^/CD62L^low^ expression on the cell surface, whereas the splenic T cells from WT mice exhibited the CD44^low^/CD62L^high^ phenotype characteristic of naïve T cells (Fig 7A and 7B), as expected. The lymph nodes from TRAF6[L74H] mice showed a similar increase in the activated CD8 and CD4 cell phenotype (Fig 7C and 7D). The change in expression of these cell surface markers in the TRAF6[L74H] splenic T cells was accompanied by an increase in Forward Scatter (FSC), a measure of cell size, and Side Scatter (SSC), a measure of cell granularity (Fig 7E).

### Elevated numbers of splenic T regulatory cells in TRAF6[L74H] mice

T regulatory cells (Tregs) are a CD4^+^ lineage T cell defined by the expression of the transcription factor FoxP3 and the CD4^+^ co-receptor CD25. They have a critical role in restricting the activation of effector T cells, as shown by the development of autoimmunity and multi-organ inflammation in “Scurfy” mice, which lack Tregs [20, 21].

We found that the spleens of TRAF6[L74H] mice expressed increased levels of CD4^+^CD25^+^FOXP3^+^ Tregs relative to WT spleens (Fig 7F and S4A Fig), similar to observations made in mice with a specific deletion of TRAF6 in Tregs [22].

### T follicular helper (Tfh cells)

Germinal Centre B (GCB) cell activation requires the activation of CD4^+^ T follicular helper (Tfh) cells, which migrate to the lymph nodes to activate cognate B cells. Consistent with the high levels of isotype-switched antibodies and autoantibodies in the serum of TRAF6[L74H] mice, we observed increased numbers of splenic Tfh cells, defined by the expression of the surface markers PD1 and CXCR5 (Fig 7G, S4B Fig).

The inducible T cell co-stimulator (ICOS) is another defining marker of Tfh cells, which is induced during T cell activation to maintain the Tfh cell phenotype [23, 24]. ICOS induces inactivation of the transcription factor FOXO1, suppressing the expression of KLF2 and sustaining surface expression of other essential Tfh markers, like CXCR5 [24]. Sanroque mice, in which the ICOS repressor is dysregulated, exhibit autoimmunity [25, 26]. We observed that ICOS expression levels were also elevated in the Tfh cells from TRAF6[L74H] spleens (S4C Fig).

### Cytokine levels in the serum

**Fig 8:**
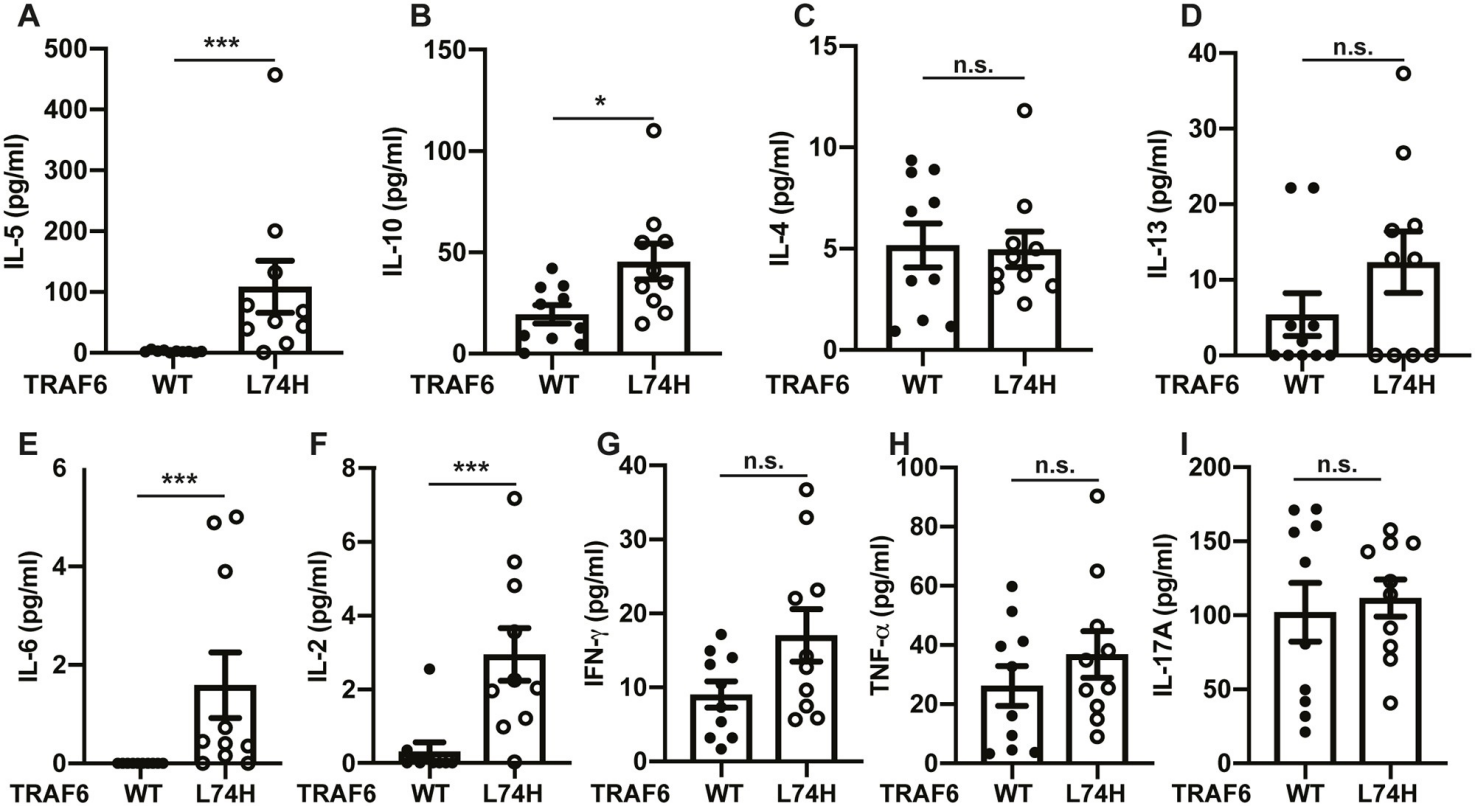
Serum cytokine levels in TRAF6[L74H] mice. (A-I) Bar graphs showing serum concentrations of IL-5 (A), IL-10 (B), IL-4 (C), IL-13 (D), IL-6 (E), IL-2 (F), IFN-γ (G), TNF-α (H), and IL-17A (I). The error bars show ± SEM. Symbols represent individual biological replicates. Statistical significance between the two genotypes was calculated using the unpaired t-test with Welch’s correction (IL-10, IFN-γ and TNF-α) or the Mann-Whitney test (IL-4, IL-5, IL-13, IL-6, IL-2 and IL-17A). * denotes p<0.05, ** denotes p<0.01, *** denotes p<0.001 and n.s. denotes not significant difference. Individual values, descriptive statistics and results from the statistical analysis are provided in S8 File.

The high levels of serum immunoglobulins, especially IgA, IgG1 and IgE, were suggestive of class-switch recombination events in the B cells of TRAF6[L74H] mice that are stimulated by CD4^+^ helper T2 (Th2) cells. We therefore measured the serum levels of a number of cytokines in 16-day mice that are produced by Th2 cells, such as IL-4, IL-5 and IL-13 and the anti-inflammatory cytokine IL-10. Consistent with a Th2 response, there were elevated levels of both IL-5 and IL-10 in the serum of TRAF6[L74H] mice (Fig 8A and 8B), but no difference in the level of IL-4 was observed (Fig 8C). IL-13 levels were increased in some, but not all of these mice (Fig 8D).

IL-6 was elevated in the serum of TRAF6[L74H] mice (Fig 8E), whose functions include the promotion of Th2 differentiation [27]. However IL-2, a Th1 cytokine, was also elevated (Fig 8F) and increases in the Th1 cytokine interferon γ (IFNγ) were seen in some mice (Fig 8G). There was little difference in the serum levels of TNFα or IL-17 (Fig 8H and 8I).

### Thymocyte development

The process of negative selection of thymocytes that recognise self-peptides with high affinity is an essential mechanism by which the immune system achieves tolerance and prevents autoimmunity. TRAF6 KO mice display an abnormal thymic architecture with reduced populations of medullary Thymic Epithelial Cells (mTECs) and their developmental precursors [28]. The mTECs are a unique population of stromal cells, which are essential for the establishment of central tolerance, and knock-out mice in which the TRAF6 gene is disrupted specifically in mTECs exhibit an abnormal thymic architecture and display high levels of autoantibodies in the serum [28, 29].

We therefore compared the thymocyte populations of TRAF6[L74H] and wild type mice but failed to detect any abnormalities in the thymocyte populations of TRAF6[L74H] mice. The weight of the thymus (S5A Fig), the total number of thymocytes (S5B Fig) and the different thymic subsets (S5C–S5F Fig) did not differ significantly between WT and TRAF6[L74H] mice at 16 days. The populations of T Cell Receptor β (TCRβ) low and the total TCRβ high thymocytes were comparable in TRAF6[L74H] and WT mice (S5C Fig). The populations of TCRβ^+^ CD4 and CD8 double negative, double positive and single positive (CD4^+^ or CD8^+^) thymocytes (S5E and S5F Fig) were also similar in TRAF6[L74H] and WT mice. Consistent with the above observations, no differences in the thymic architecture (S6A and S6B Fig), or CD3^+^ and IBA-1^+^ staining were observed between the TRAF6[L74H] and WT littermate control mice (S6C–S6F Fig).

### Heterozygous TRAF6[L74H] mice

In contrast to homozygous TRAF6[L74H] mice, heterozygous mice did not display any flaking and scaling of the skin even when they were 17–21 weeks old and their grimace scores did not differ from age matched wild mice (S7A Fig). The spleen (S7B Fig) and lymph nodes (S7C Fig) were not enlarged, and no differences in the numbers of splenic immune cells were detected compared to WT mice (S7D–S7K Fig). Moreover, the numbers of CD4^+^ and CD8^+^ T cells, as well as the proportion of CD44^high^ CD62^low^ T cells compared to CD44^low^CD62^high^ T cells were similar to WT and characteristic of naïve T cells (S7L–S7Q Fig). The levels of immunoglobulins measured in the serum of heterozygous mice were similar to WT levels (S8A–S8F Fig), apart from a small increase in IgM in the heterozygous mice (S8G Fig). Therefore 50% of the normal level of TRAF6 E3 ligase activity is sufficient to prevent the autoimmune/autoinflammatory phenotype from developing.

## Discussion

The widespread application of mouse knock-out technology has greatly advanced our understanding of the physiological roles of many proteins. However, the molecular mechanisms underlying the phenotypes of knock-out mice or cells derived from them can be difficult to interpret because, in the case of enzymes, eliminating the expression of the entire protein removes every functional domain and not just the catalytic activity. The complete absence of a protein may prevent its interaction with other proteins, which may change the stability of the interacting proteins causing their level(s) to increase or decrease, so that the phenotypic changes observed may be indirect. Moreover, in the complete absence of a protein, its functions may be replaced by another protein(s), which may obscure, exacerbate or suppress the phenotype, and lead to erroneous conclusions being reached about the function of the enzymatic activity in vivo. For these reasons, the generation of knock-in mice in which the catalytic function of an enzyme is ablated by a single amino acid substitution can provide more definitive information about the functions of the catalytic activity in vivo.

TRAF6 KO mice lacking TRAF6 in every cell of the body have been produced in several laboratories [1–6] and studied extensively for over 20 years. More recently, we generated TRAF6[L74H] mice in which only the E3 ligase activity of TRAF6 was ablated [12], but all other functional domains and expression of the protein were preserved. We found that, in contrast to TRAF6 KO mice, which have abnormal bones and lack teeth, the long bones of the TRAF6[L74H] mice were normal histologically and their teeth erupted normally. Here we have extended these observations considerably and shown that, remarkably, TRAF6[L74H] mice do not display any of the major phenotypes of mice lacking TRAF6 expression in every cell of the body. In particular, and in contrast to TRAF6 KO mice, the TRAF6[L74H] mice have normal sebaceous glands, they express guard hair follicles, their lymph nodes are not absent but enlarged, and their B cells are not decreased.

Most, if not all, of the phenotypic differences between TRAF6 KO mice and wild type mice appear to be explained by the absence of TNF receptor superfamily (TNFRSF) signalling in TRAF6 KO mice, which is either not impaired, or only partially impaired, in TRAF6[L74H] mice. For example, the TNFRSF member RANK is known to be required for the development of lymph nodes [30], in addition to its role in osteoclast development, the TNFRSF members TROY and XEDAR are required for the development of guard-hair follicles [1], while the TNFRSF member CD40 has a critical role in Memory B cell development, the formation of GCB cells [31], and the maturation, activation and development of dendritic cells [5]. The present study implies that although the expression of the TRAF6 protein is essential for signal transduction by RANKL, TROY, XEDAR and CD40, the E3 ligase activity of TRAF6 is dispensable, since the major essential functions of these TNFRSF members seem to be preserved in mice expressing the E3 ligase-inactive TRAF6[L74H] mutant.

Although the TRAF6[L74H] mice did not develop the phenotypes displayed by mice with a knock-out of TRAF6 in every cell of the body, they instead developed autoimmunity and multi-organ inflammation within 16 days of birth, phenotypes that have not been reported in TRAF6 KO mice that survive to this age. The reason why mice lacking TRAF6 expression in every cell do not develop autoimmunity and autoinflammation may be explained by their lack of lymph nodes and GCB cells, and the impaired maturation and activation of their dendritic cells, which appear to be consequences of the absence of TNFRSF signalling in these mice.

The phenotype of TRAF6[L74H] mice resembles quite closely the phenotype of mice that do not express TRAF6 in T regulatory cells (Tregs). It is also similar to the phenotype of mice with a specific KO of TRAF6 in all T cells and in which the Tregs are non-functional in vivo [32, 33]. Like the TRAF6[L74H] mice, mice lacking TRAF6 in Tregs or all T cells have enlarged lymph nodes, display splenomegaly, generate GCB cells spontaneously, have elevated serum levels of a variety of antibodies and develop autoimmunity and multiorgan inflammation including dermatitis [22].

The hallmark of Tregs is their expression of FoxP3 but, curiously, the number of FoxP3^+^ Tregs in the spleen and lymph nodes of mice with a Treg-specific KO of TRAF6 was increased 2 to 3-fold, even though they are non-functional [22]. We observed a similar increase in the number of splenic FoxP3^+^ Tregs in TRAF6[L74H] mice compared to WT mice (Fig 7A and S3A Fig). These observations indicate that TRAF6 and its E3 ligase activity are not required for the expression of FoxP3 in Tregs. It is possible that the increased numbers of Tregs in mice with a Treg specific KO of TRAF6 and in TRAF6[L74H] mice may reflect an attempt by the Tregs to compensate for their loss of functionality. Mice with a Treg-specific KO of TRAF6 have a much higher proportion of CD44^high^ CD62L^low^ CD4^+^ effector T cells in their spleen and lymph nodes [22]. These studies indicated that the Tregs in these mice are unable to restrict the activation of CD4^+^ effector T cells and are therefore non-functional. Here, we found that the TRAF6[L74H] mice also have a much higher proportion of CD44^high^ CD62L^low^ CD4^+^ and CD8^+^ effector T cells in their spleen. Taken together, these observations suggest that Tregs expressing the E3 ligase-inactive TRAF6[L74H] mutant may also be non-functional and unable to restrict the activation of effector CD4^+^ and CD8^+^ T cells.

An obvious difference between TRAF6[L74H] knock-in mice and mice lacking TRAF6 expression in Tregs or all T cells is the speed at which the phenotype develops. The TRAF6[L74H] mice develop autoimmunity, skin and liver inflammation within 16 days, while mice with a specific knock-out of TRAF6 in Tregs [33] or all T cells [32, 33] do not develop these phenotypes until they are 8–11 weeks old. This difference could be due to the distinct microbiomes of these mice, but it may also be caused by impairment of other, non-T cell functions in TRAF6[L74H] mice which accelerate the development of the phenotype. For example, the severe skin inflammation and hyperkeratosis observed in TRAF6[L74H] mice has not been reported in mice with a specific knock-out of TRAF6 in Tregs [33] or all T cells [32, 33]. Moreover, the outermost skin cells of TRAF6[L74H] mice display enhanced senescence compared to WT mice (S2C Fig), which may be driven by hyperkeratosis and lead to the observed flaking of the skin. The increased number of dying ectodermal cells in TRAF6[L74H] mice may also cause secondary inflammations that contribute to the accelerated rate at which the autoinflammatory phenotype develops [34–37] compared to mice with a specific knock-out of TRAF6 in Tregs [33] or in all T cells [32, 33].

Cytotoxic T cells recognize and destroy cancer cells, but their activation in WT mice is constrained by a number of inhibitory mechanisms. Ipilimumab, an antibody that relieves the inhibition of cytotoxic T cells by targeting CTLA-4, has achieved “blockbuster” status, since its approval for melanoma and lung cancer in 2011. Interestingly, the phenotype of CTLA-4 KO mice resembles that of TRAF6[L74H] mice [38]. The present study therefore raises the possibility that a drug inhibiting the TRAF6 E3 ligase activity may also unleash the power of cytotoxic T cells to destroy cancer cells, without developing the serious adverse effects seen in TRAF6 KO mice.

## Supporting information

S1 Fig Characterization of splenic immune cell populations in TRAF6[L74H] mice. **(A, B)** Splenocytes isolated from 16-day old WT and TRAF6[L74H] were stained with DAPI, anti-CD19, anti-TCRβ, anti-CD4, anti-CD8, anti-CD44 and anti-CD62L antibodies. Cells were gated based on FSC-A and SSC-A, doublets were excluded and DAPI^-^ live cells were further analyzed. (**A**) Representative flow cytometry plots showing the expression of CD19 and TCRβ in DAPI^-^ live cells and the proportion of CD8 and CD4 T cells within the TCRβ^+^ cells. (**B**) Total number of CD8 T cells and CD4 T cells in the spleens of WT (n = 8) and TRAF6[L74H] (n = 9) mice. Symbols represent individual biological replicates. Significance between the two genotypes was calculated using the student’s t-test; n.s. indicates that differences were not significant. (**C**) As in A, except representative flow cytometry plots shows the percentage of GR-1^hi^ CD11b^+^ (neutrophils) and GR-1^lo-med^ CD11b^+^ (myeloid cells). (**D**) As in A, except that representative flow cytometry plots show the percentage of Ter119^+^ cells (erythrocytes). Individual values, descriptive statistics and results from the statistical analysis are provided in S9 File.
(TIF)

S2 Fig p21 and TUNEL staining of the skin of TRAF6[L74H] mice. **(A, B)** Representative images of skin sections from the ears stained with an anti-p21 antibody from WT (**A**) and TRAF6[L74H] (**B**) mice. (**C**) Bar graphs showing semi-quantitative scores of the ear epidermis layer. (**D**) Image of an ear skin section from one WT mouse and one TRAF6[L74H] mouse stained with TUNEL to detect dead cells (green) and with DAPI to detect nuclei (blue). (**E**) Quantitation of the percentage TUNEL positive cells relative to the total number of DAPI positive cells. Circles represent individual biological replicates. The circles highlighted in red in E correspond to the WT and TRAF6[L74H] mice shown in D. Significance between the two genotypes was calculated using the Mann-Whitney Test. * denotes p<0.05. Individual values, descriptive statistics and results from the statistical analysis are provided in S10 File.
(TIF)

S3 Fig Enhanced PAX5+ staining in the liver and lung of TRAF6[L74H] mice. **(A, B)** Representative immunohistochemistry image of liver sections processed for staining with anti-PAX5 antibody from WT (**A**) and TRAF6[L74H] (**B**) mice. (**C, D**) As in A, B except that lungs sections were processed.
(TIF)

S4 Fig Increase in Tregs and T_FH_ cells in TRAF6[L74H] knock-in mice. **(A)** Splenocytes isolated from 16-day old WT and TRAF6[L74H] mice were stained with anti-TCRβ, anti-CD4, anti-CD25 and anti-FoxP3 antibodies. The representative flow cytometry plots show the percentage of CD25^+^FoxP3^+^ Treg cells from the TCRβ^+^CD4^+^ population. (**B, C**) As in (**A**), except that splenic cells were stained with anti-TCRβ, anti-CD4, anti-PD-1, anti-CXCR5, anti-ICOS and anti-Bcl6 antibodies. (**B**) Plots show the % of PD-1^+^CXCR5^+^ T_FH_ cells from the TCRβ^+^CD4^+^ population. (**C**) Representative histograms showing the expression of ICOS and Bcl6 in the TCRβ^+^CD4^+^ PD-1^+^CXCR5^+^ population in WT and TRAF6[L74H] mice.
(TIF)

S5 Fig T cell development in the thymus is normal in TRAF6[L74H] knock-in mice. (**A**) Thymus weight in 16-day old WT and TRAF6[L74H] knock in mice. (**B**) Total number of cells in the thymus of WT and TRAF6[L74H] knock in mice. (**C**) Thymic cells were stained with DAPI, anti-TCRβ, anti-CD4 and anti-CD8 antibodies. Cells were gated based on FSC-A and SSC-A, doublets were excluded and DAPI^-^ live cells were further analyzed. The representative flow cytometry plots show the percentage of TCRβ^low^ and TCRβ^high^ cells from all live thymocytes. (**D**) As in C, except that total numbers of TCRβ^low^ and TCRβ^high^ cells are shown. (**E**) Representative plots showing the expression of CD4 and CD8 in the DAPI^-^TCRβ^high^ population. (**F**) Plots showing the total numbers of double negative (DN) DAPI^-^TCRβ^high^CD4^-^CD8^-^, double positive (DP) DAPI^-^TCRβ^high^CD4^+^CD8^+^, CD8 single positive (SP) DAPI^-^TCRβ^high^CD4^-^CD8^+^ and CD4 SP DAPI^-^TCRβ^high^CD4^+^CD8^-^ T cells in the thymus of WT and TRAF6 [L74H] mice. In A, B, D and F symbols represent individual biological replicates. Significance between the two genotypes was calculated using the Student T-test or Mann-Whitney test; n.s., not significantly different. Individual values, descriptive statistics and results from the statistical analysis are provided in S11 File.
(TIF)

S6 Fig Thymic architecture is not perturbed in TRAF6[L74H] mice. **(A, B)** Representative image showing haematoxylin and eosin (H & E)-stained thymic sections from WT (**A**) and TRAF6[L74H] (**B**) mice. (**C, D**) As in **A**, **B** except that thymic sections were processed for immunohistochemistry staining using anti-CD3 antibody. (**E, F**) As in **C, D** except that anti-IBA-1 antibody was used.
(TIF)

S7 Fig Characterization of the phenotype of TRAF6[L74H] heterozygous mice. (**A**) Body weights of 18–21 week old WT (n = 4) and TRAF6[L74H] heterozygous (n = 3) male mice **(B**) Spleen weight (left panel) and representative images of spleen size (right panel) of 18–21 week old WT (n = 4) and TRAF6[L74H] heterozygous (n = 4) mice, scale bar = 1 cm. (**C**) Representative images of axillary and brachial lymph nodes (left panel) and inguinal lymph nodes (right panel) of one WT and one TRAF6[L74H] heterozygous mouse. (**D**) Splenocyte numbers of 18–21 week WT (n = 4) and TRAF6[L74H] heterozygous (n = 4) mice. (**E-Q**) As in D, except that splenic immune cell populations were analyzed by flow cytometry. Plots show total numbers of B cells (**E**), T cells (**F**), non-T/B cells (**G**), neutrophils (**H**), myeloid cells (**I**), Treg cells (**J**) and Tfh cells (**K**). Total numbers of CD4 T cells (**L**), percentage of CD44^+^ (**M**) and CD62L^+^ (**N**) from all CD4 T cells and total numbers of CD8 T cells (**O**) and the percentage of CD44^+^ (**P**) and CD62L^+^ (**Q**) from all CD8 T cells are shown. Symbols represent individual biological replicates. Statistical significance between the two genotypes was calculated using the unpaired t-test with Welch’s correction; n.s. denotes not significant difference. Individual values, descriptive statistics and results from the statistical analysis are provided in S12 File.
(TIF)

S8 Fig Serum immunoglobulin levels in WT and TRAF6[L74H] heterozygous mice. Concentrations of IgA (**A**), IgG1 (**B**), IgG2b (**C**), IgG2a (**D**), IgG3 (**E**), IgE (**F**) and IgM (**G**) in the serum of 18–21 week WT (n = 4) and TRAF6[L74H] heterozygous (n = 4) mice. Symbols represent individual biological replicates. Statistical significance between the two genotypes was calculated using the unpaired t-test with Welch’s correction; n.s. denotes that the difference is not significant. * denotes p<0.05. Individual values, descriptive statistics and results from the statistical analysis are provided in S13 File.
(TIF)

S1 File Individual values and statistical analysis of the data shown in Fig 1. (XLSX)

S2 File Individual values and statistical analysis of the data shown in Fig 2. (XLSX)

S3 File Individual values and statistical analysis of the data shown in Fig 3. (XLSX)

S4 File Individual values and statistical analysis of the data shown in Fig 4. (XLSX)

S5 File Individual values and statistical analysis of the data shown in Fig 5. (XLSX)

S6 File Individual values and statistical analysis of the data shown in Fig 6. (XLSX)

S7 File Individual values and statistical analysis of the data shown in Fig 7. (XLSX)

S8 File Individual values and statistical analysis of the data shown in Fig 8. (XLSX)

S9 File Individual values and statistical analysis of the data shown in S1 Fig. (XLSX)

S10 File Individual values and statistical analysis of the data shown in S2 Fig. (XLSX)

S11 File Individual values and statistical analysis of the data shown in S5 Fig. (XLSX)

S12 File Individual values and statistical analysis of the data shown in S7 Fig. (XLSX)

S13 File Individual values and statistical analysis of the data shown in S8 Fig. (XLSX)
