# Supplementary figures and images for: Why are the phenotypes of TRAF6 knock-in and TRAF6 knock-out mice so different?
Source: PLoS One. 2022 Feb 14;17(2):e0263151. doi: 10.1371/journal.pone.0263151 (PMC8843210; doi:10.1371/journal.pone.0263151)

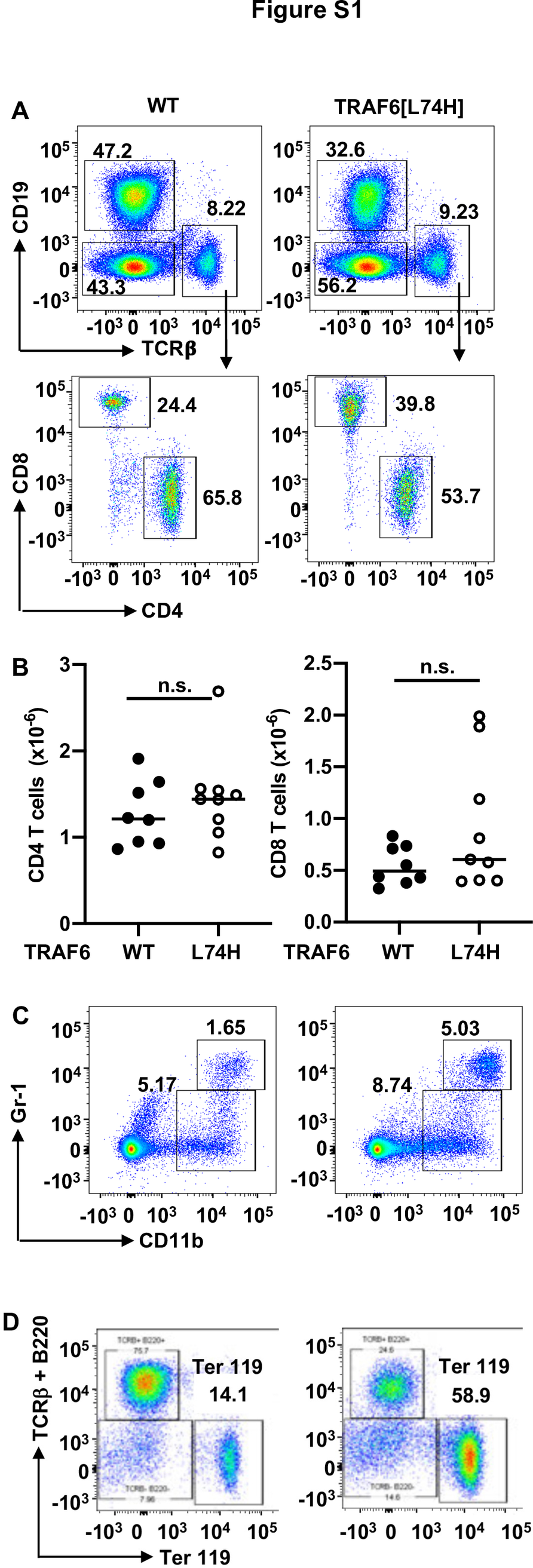

Supplement: S1 Fig — (A, B) Splenocytes isolated from 16-day old WT and TRAF6[L74H] were stained with DAPI, anti-CD19, anti-TCRβ, anti-CD4, anti-CD8, anti-CD44 and anti-CD62L antibodies. Cells were gated based on FSC-A and SSC-A, doublets were excluded and DAPI- live cells were further analyzed. (A) Representative flow cytometry plots showing the expression of CD19 and TCRβ in DAPI- live cells and the proportion of CD8 and CD4 T cells within the TCRβ+ cells. (B) Total number of CD8 T cells and CD4 T cells in the spleens of WT (n = 8) and TRAF6[L74H] (n = 9) mice. Symbols represent individual biological replicates. Significance between the two genotypes was calculated using the student’s t-test; n.s. indicates that differences were not significant. (C) As in A, except representative flow cytometry plots shows the percentage of GR-1hi CD11b+ (neutrophils) and GR-1lo-med CD11b+ (myeloid cells). (D) As in A, except that representative flow cytometry plots show the percentage of Ter119+ cells (erythrocytes). Individual values, descriptive statistics and results from the statistical analysis are provided in S9 File. (TIF) [file pone.0263151.s001.tif]

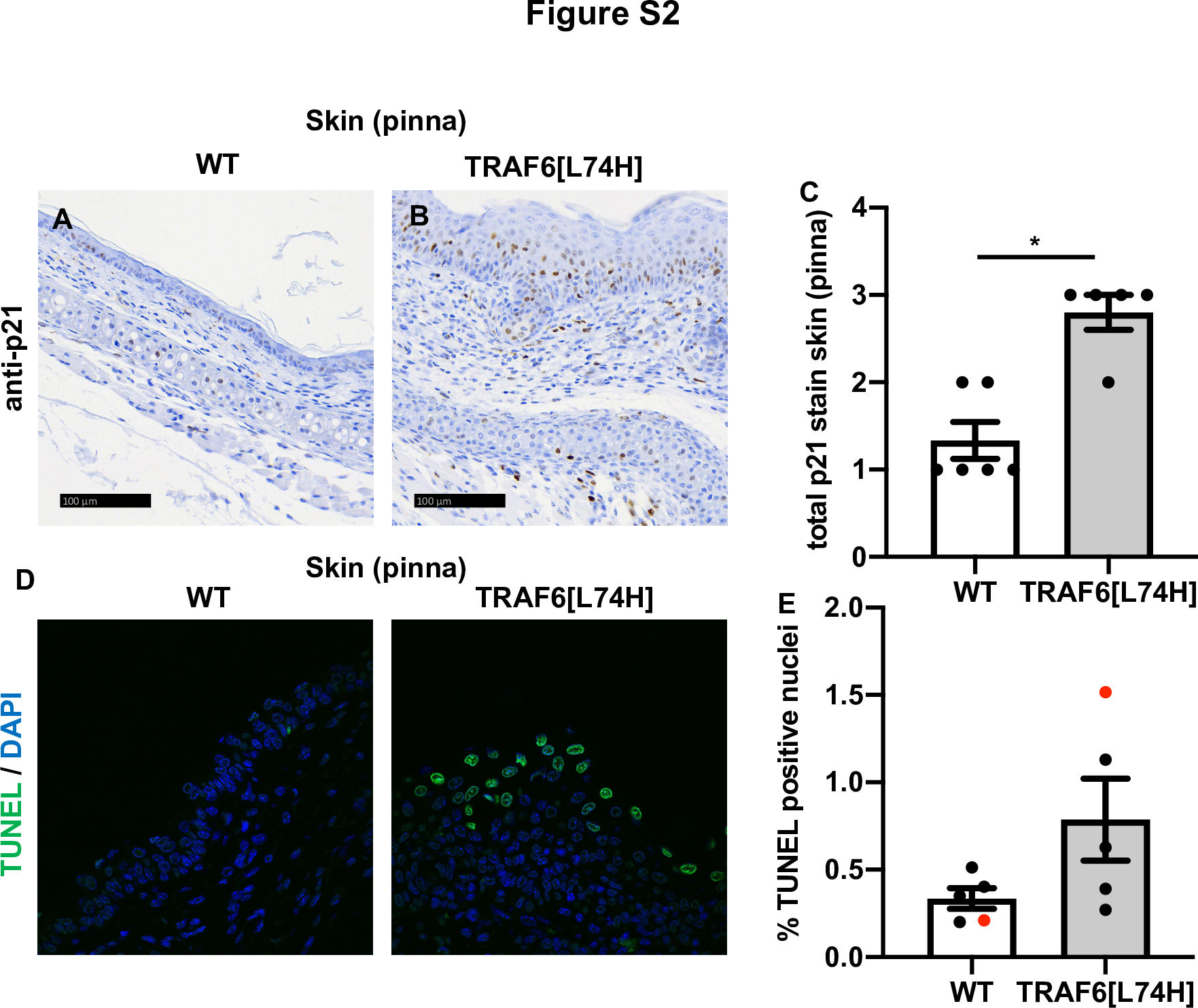

Supplement: S2 Fig — (A, B) Representative images of skin sections from the ears stained with an anti-p21 antibody from WT (A) and TRAF6[L74H] (B) mice. (C) Bar graphs showing semi-quantitative scores of the ear epidermis layer. (D) Image of an ear skin section from one WT mouse and one TRAF6[L74H] mouse stained with TUNEL to detect dead cells (green) and with DAPI to detect nuclei (blue). (E) Quantitation of the percentage TUNEL positive cells relative to the total number of DAPI positive cells. Circles represent individual biological replicates. The circles highlighted in red in E correspond to the WT and TRAF6[L74H] mice shown in D. Significance between the two genotypes was calculated using the Mann-Whitney Test. * denotes p<0.05. Individual values, descriptive statistics and results from the statistical analysis are provided in S10 File. (TIF) [file pone.0263151.s002.tif]

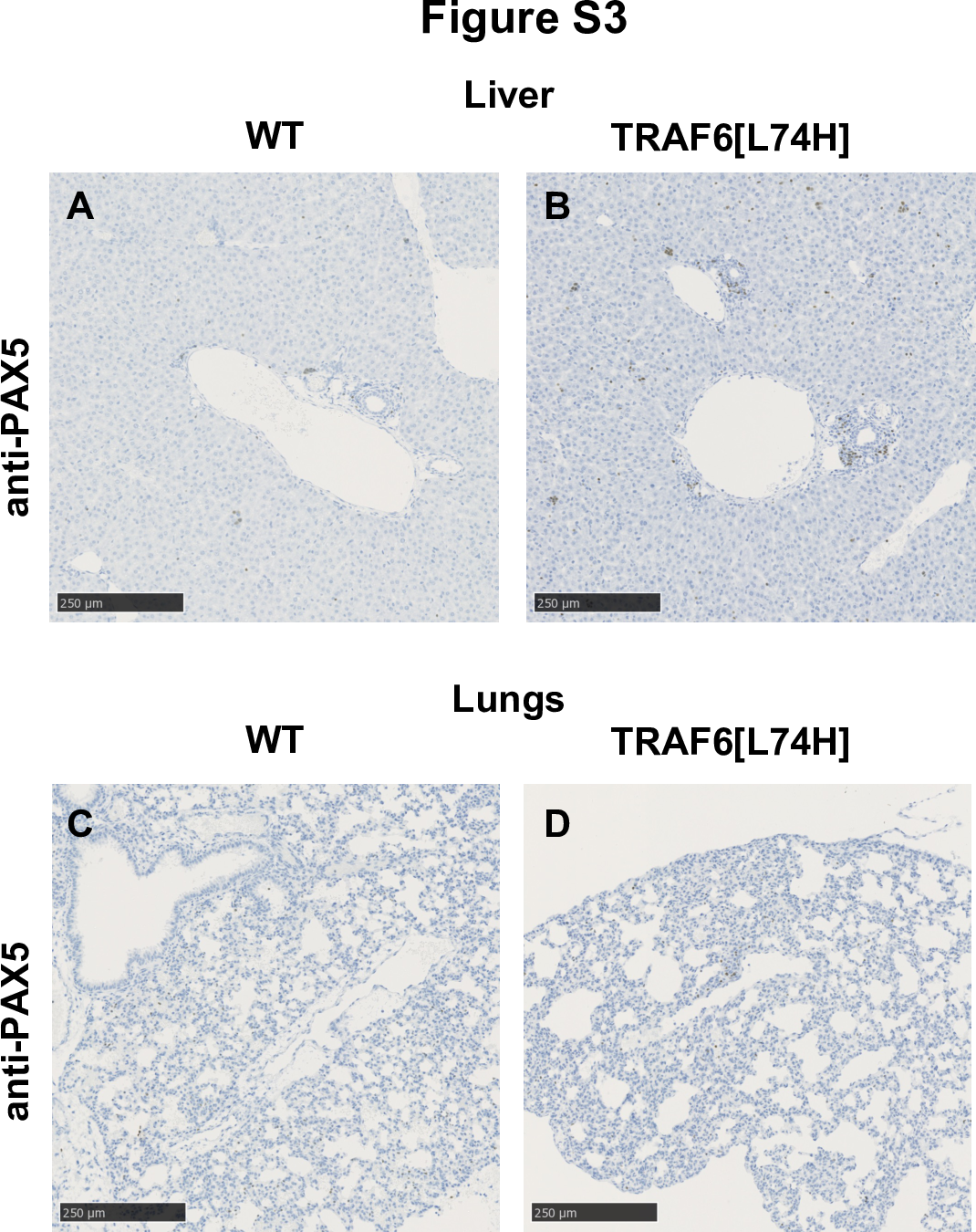

Supplement: S3 Fig — (A, B) Representative immunohistochemistry image of liver sections processed for staining with anti-PAX5 antibody from WT (A) and TRAF6[L74H] (B) mice. (C, D) As in A, B except that lungs sections were processed. (TIF) [file pone.0263151.s003.tif]

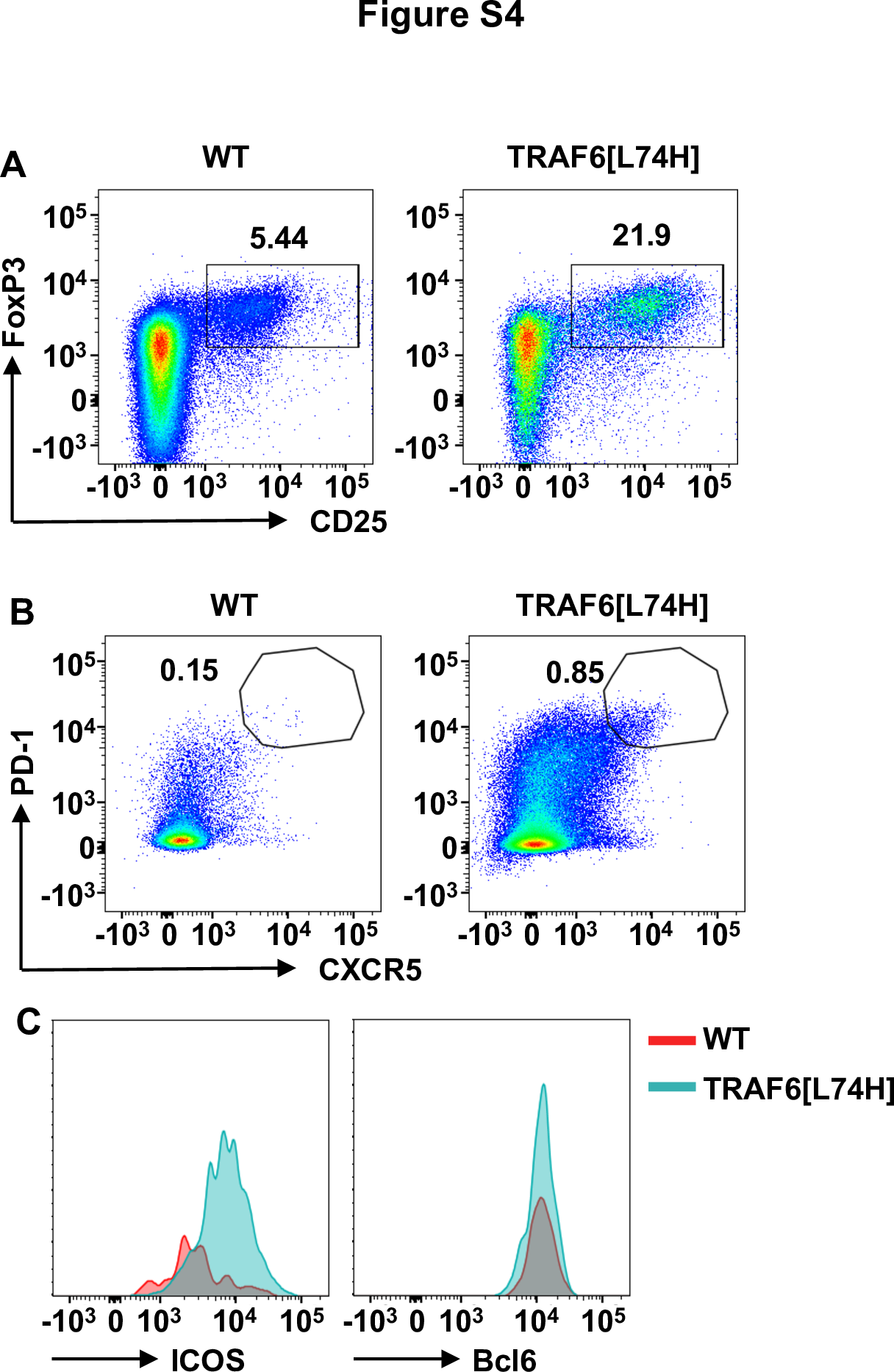

Supplement: S4 Fig — (A) Splenocytes isolated from 16-day old WT and TRAF6[L74H] mice were stained with anti-TCRβ, anti-CD4, anti-CD25 and anti-FoxP3 antibodies. The representative flow cytometry plots show the percentage of CD25+FoxP3+ Treg cells from the TCRβ+CD4+ population. (B, C) As in (A), except that splenic cells were stained with anti-TCRβ, anti-CD4, anti-PD-1, anti-CXCR5, anti-ICOS and anti-Bcl6 antibodies. (B) Plots show the % of PD-1+CXCR5+ TFH cells from the TCRβ+CD4+ population. (C) Representative histograms showing the expression of ICOS and Bcl6 in the TCRβ+CD4+ PD-1+CXCR5+ population in WT and TRAF6[L74H] mice. (TIF) [file pone.0263151.s004.tif]

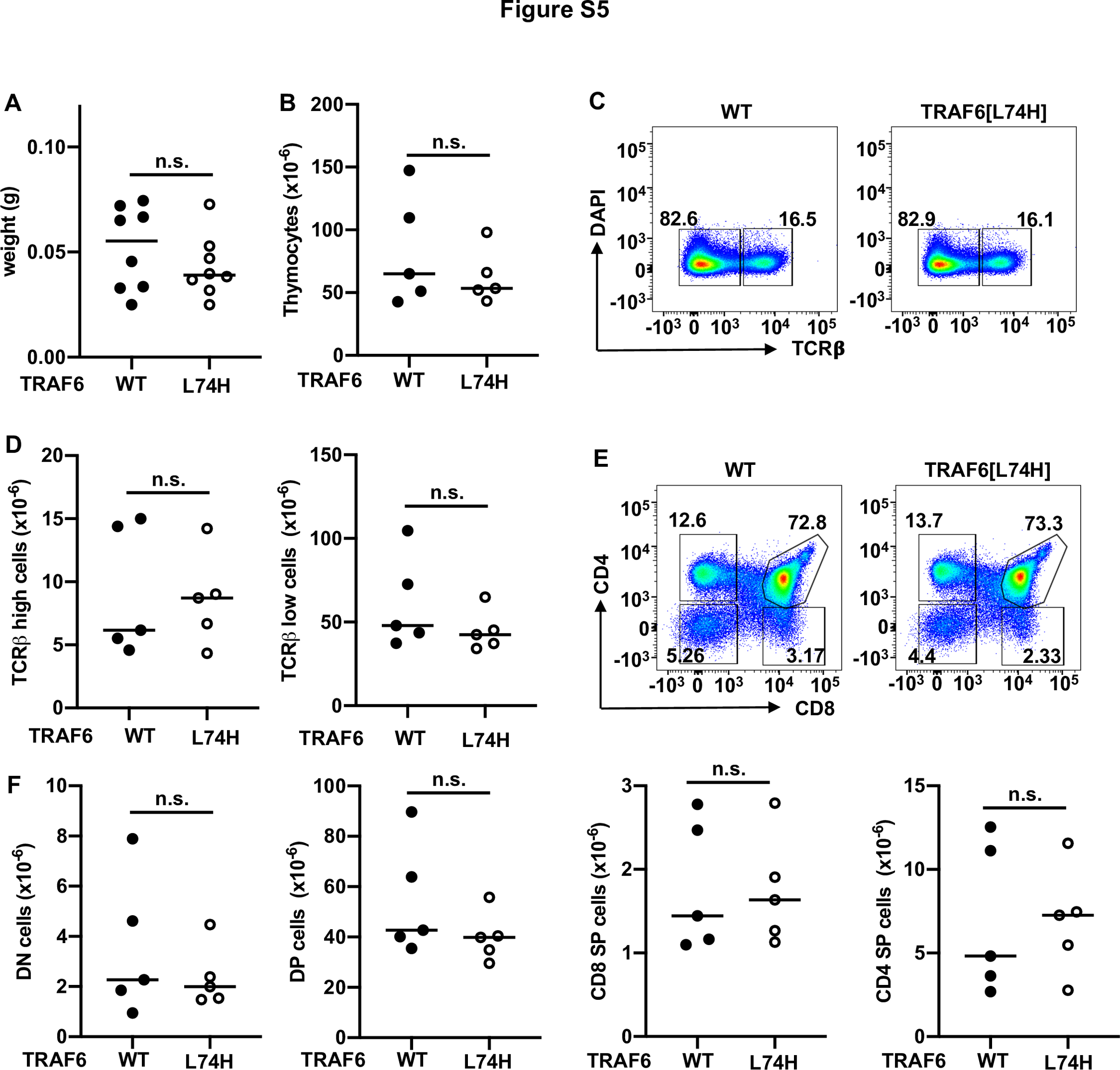

Supplement: S5 Fig — (A) Thymus weight in 16-day old WT and TRAF6[L74H] knock in mice. (B) Total number of cells in the thymus of WT and TRAF6[L74H] knock in mice. (C) Thymic cells were stained with DAPI, anti-TCRβ, anti-CD4 and anti-CD8 antibodies. Cells were gated based on FSC-A and SSC-A, doublets were excluded and DAPI- live cells were further analyzed. The representative flow cytometry plots show the percentage of TCRβlow and TCRβhigh cells from all live thymocytes. (D) As in C, except that total numbers of TCRβlow and TCRβhigh cells are shown. (E) Representative plots showing the expression of CD4 and CD8 in the DAPI-TCRβhigh population. (F) Plots showing the total numbers of double negative (DN) DAPI-TCRβhighCD4-CD8-, double positive (DP) DAPI-TCRβhighCD4+CD8+, CD8 single positive (SP) DAPI-TCRβhighCD4-CD8+ and CD4 SP DAPI-TCRβhighCD4+CD8- T cells in the thymus of WT and TRAF6 [L74H] mice. In A, B, D and F symbols represent individual biological replicates. Significance between the two genotypes was calculated using the Student T-test or Mann-Whitney test; n.s., not significantly different. Individual values, descriptive statistics and results from the statistical analysis are provided in S11 File. (TIF) [file pone.0263151.s005.tif]

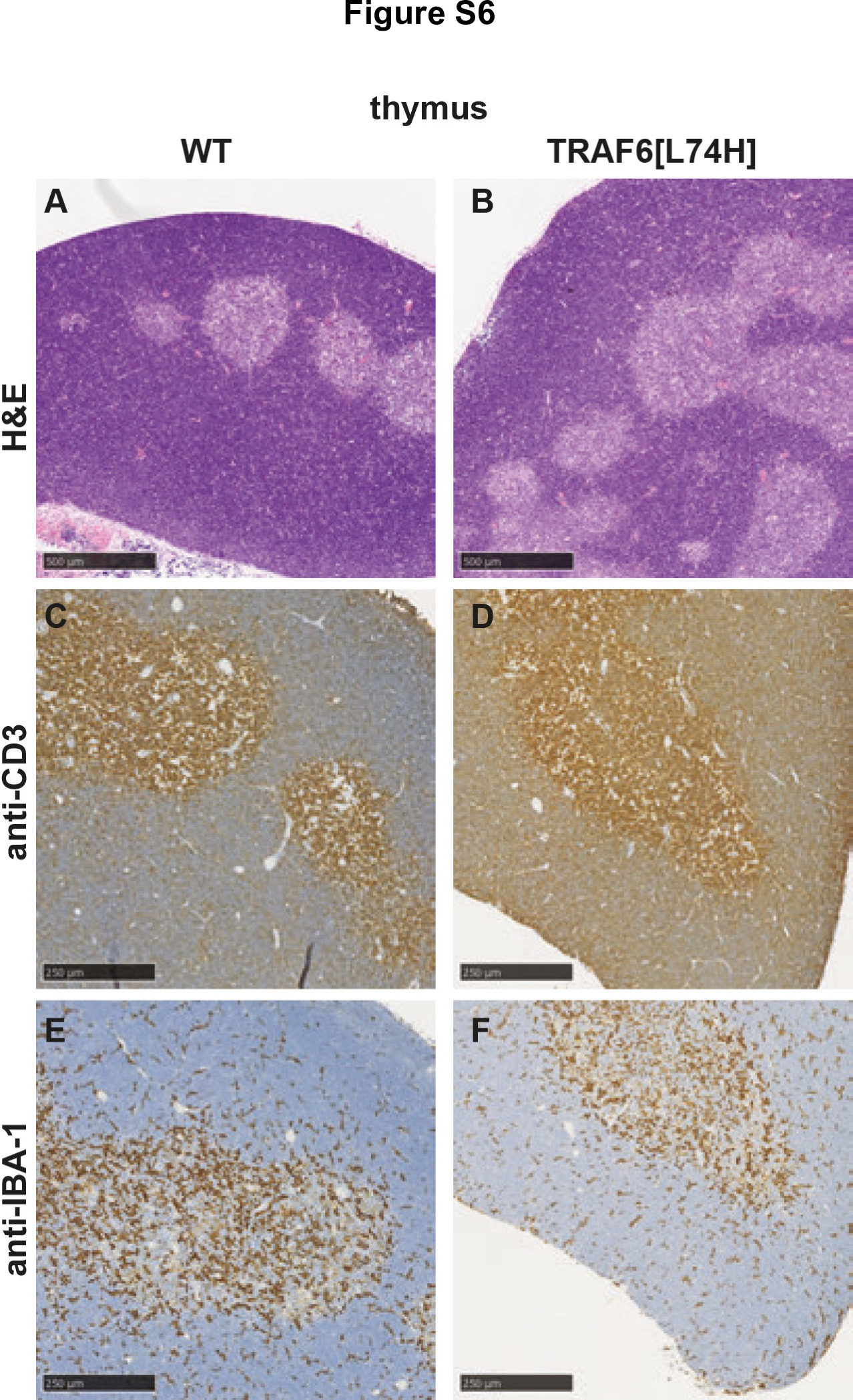

Supplement: S6 Fig — (A, B) Representative image showing haematoxylin and eosin (H & E)-stained thymic sections from WT (A) and TRAF6[L74H] (B) mice. (C, D) As in A, B except that thymic sections were processed for immunohistochemistry staining using anti-CD3 antibody. (E, F) As in C, D except that anti-IBA-1 antibody was used. (TIF) [file pone.0263151.s006.tif]

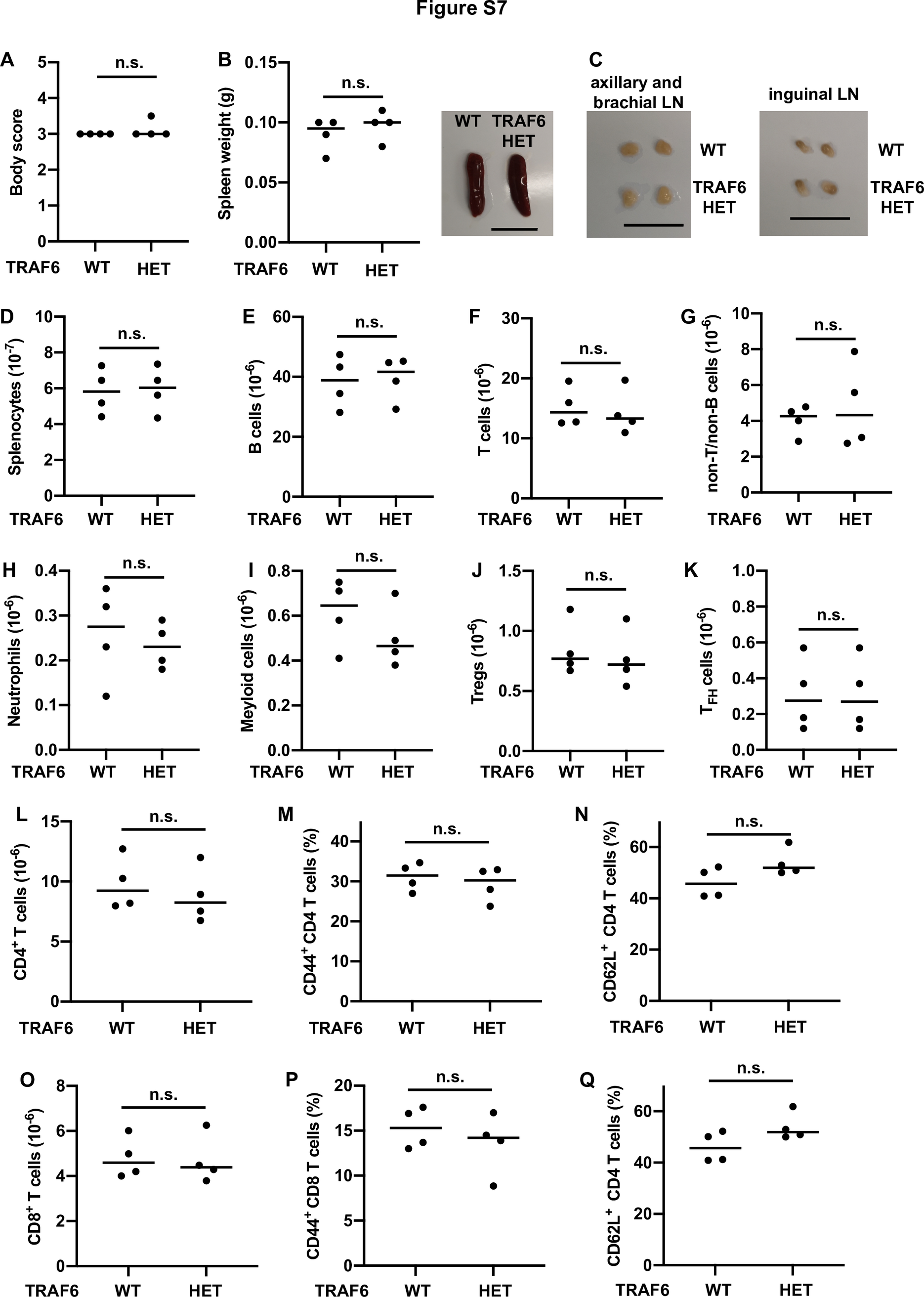

Supplement: S7 Fig — (A) Body weights of 18–21 week old WT (n = 4) and TRAF6[L74H] heterozygous (n = 3) male mice (B) Spleen weight (left panel) and representative images of spleen size (right panel) of 18–21 week old WT (n = 4) and TRAF6[L74H] heterozygous (n = 4) mice, scale bar = 1 cm. (C) Representative images of axillary and brachial lymph nodes (left panel) and inguinal lymph nodes (right panel) of one WT and one TRAF6[L74H] heterozygous mouse. (D) Splenocyte numbers of 18–21 week WT (n = 4) and TRAF6[L74H] heterozygous (n = 4) mice. (E-Q) As in D, except that splenic immune cell populations were analyzed by flow cytometry. Plots show total numbers of B cells (E), T cells (F), non-T/B cells (G), neutrophils (H), myeloid cells (I), Treg cells (J) and Tfh cells (K). Total numbers of CD4 T cells (L), percentage of CD44+ (M) and CD62L+ (N) from all CD4 T cells and total numbers of CD8 T cells (O) and the percentage of CD44+ (P) and CD62L+ (Q) from all CD8 T cells are shown. Symbols represent individual biological replicates. Statistical significance between the two genotypes was calculated using the unpaired t-test with Welch’s correction; n.s. denotes not significant difference. Individual values, descriptive statistics and results from the statistical analysis are provided in S12 File. (TIF) [file pone.0263151.s007.tif]

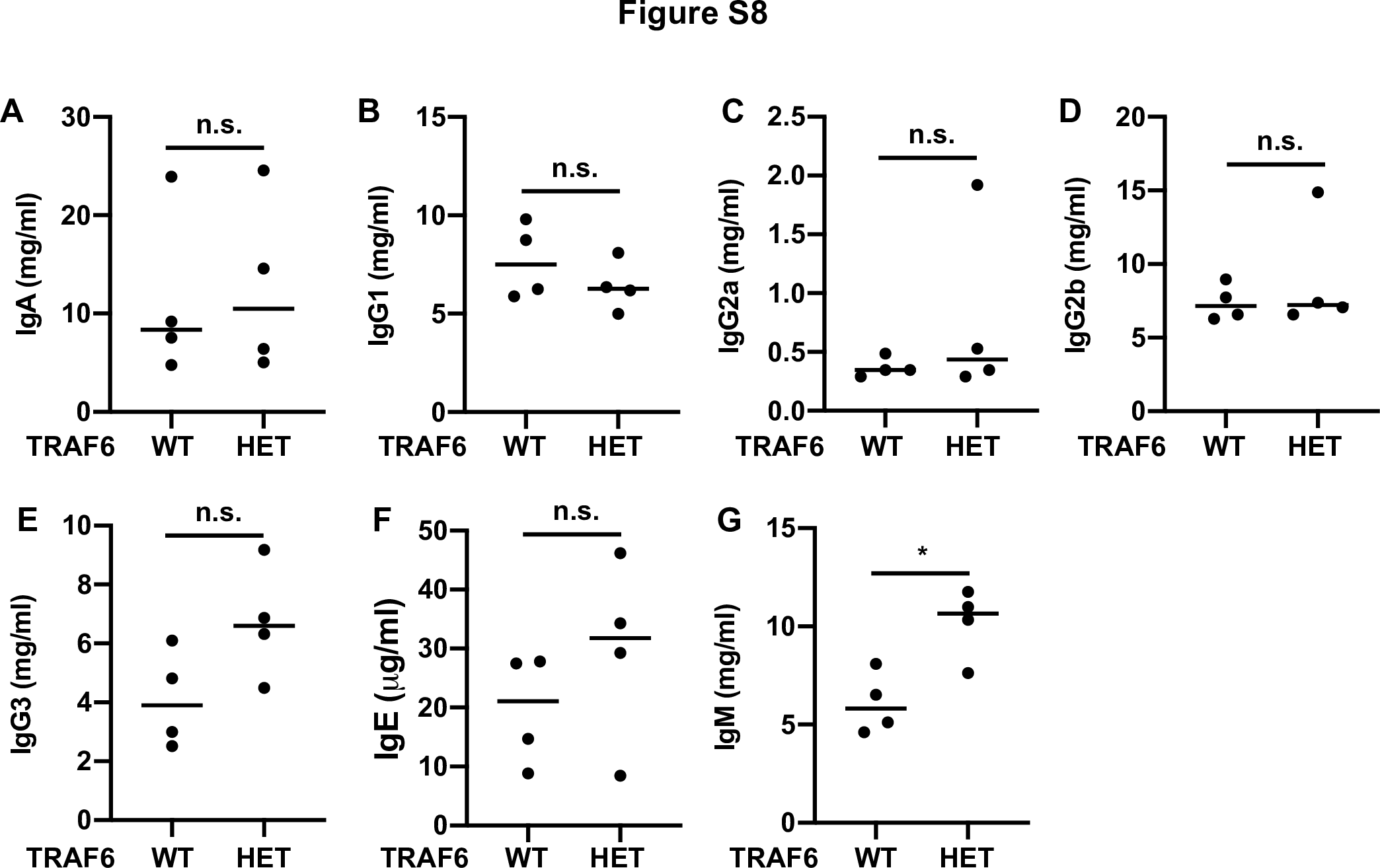

Supplement: S8 Fig — Concentrations of IgA (A), IgG1 (B), IgG2b (C), IgG2a (D), IgG3 (E), IgE (F) and IgM (G) in the serum of 18–21 week WT (n = 4) and TRAF6[L74H] heterozygous (n = 4) mice. Symbols represent individual biological replicates. Statistical significance between the two genotypes was calculated using the unpaired t-test with Welch’s correction; n.s. denotes that the difference is not significant. * denotes p<0.05. Individual values, descriptive statistics and results from the statistical analysis are provided in S13 File. (TIF) [file pone.0263151.s008.tif]
